# Assessment of Neighborhood Poverty, Cognitive Function, and Prefrontal and Hippocampal Volumes in Children

**DOI:** 10.1001/jamanetworkopen.2020.23774

**Published:** 2020-11-03

**Authors:** Rita L. Taylor, Shelly R. Cooper, Joshua J. Jackson, Deanna M. Barch

**Affiliations:** 1 Department of Psychological and Brain Sciences, Washington University, St Louis, Missouri; 2 Department of Psychiatry, Washington University, St Louis, Missouri; 3 Department of Radiology, Washington University, St Louis, Missouri

## Abstract

**Importance:**

The association between poverty and unfavorable cognitive outcomes is robust, but most research has focused on individual household socioeconomic status (SES). There is increasing evidence that neighborhood context explains unique variance not accounted for by household SES.

**Objective:**

To evaluate whether neighborhood poverty (NP) is associated with cognitive function and prefrontal and hippocampal brain structure in ways that are dissociable from household SES.

**Design, Setting, and Participants:**

This cross-sectional study used a baseline sample of the ongoing longitudinal Adolescent Brain Cognitive Development (ABCD) Study. The ABCD Study will follow participants for assessments each year for 10 years. Data were collected at 21 US sites, mostly within urban and suburban areas, between September 2019 and October 2018. School-based recruitment was used to create a participant sample reflecting the US population. Data analysis was conducted from March to June 2019.

**Main Outcomes and Measures:**

NP and household SES were included as factors potentially associated with National Institutes of Health Toolbox Cognitive Battery subtests and hippocampal and prefrontal (dorsolateral prefrontal cortex [DLPFC], dorsomedial PFC [DMPFC], superior frontal gyrus [SFG]) volumes. Independent variables were first considered individually and then together in mixed-effects models with age, sex, and intracranial volume as covariates. Structural equation modeling (SEM) was used to assess shared variance in NP to brain structure and cognitive task associations. The tested hypotheses were formulated after data collection.

**Results:**

A total of 11 875 children aged 9 and 10 years (5678 [47.8%] girls) were analyzed. Greater NP was associated with lower scores across all cognitive domains (eg, total composite: β = −0.18; 95% CI, −0.21 to −0.15; *P* < .001) and with decreased brain volume in the DLPFC (eg, right DLPFC: β = −0.09; 95% CI, −0.12 to −0.07; *P* < .001), DMPFC (eg, right DMPC: β = −0.07; 95% CI, −0.09 to −0.05; *P* < .001), SFG (eg, right SFG: β = −0.05; 95% CI, −0.08 to −0.03; *P* < .001), and right hippocampus (β = −0.04; 95% CI, −0.06 to −0.01; *P* = .01), even when accounting for household income. Greater household income was associated with higher scores across all cognitive domains (eg, total composite: β = 0.30; 95% CI, 0.28 to 0.33; *P* < .001) and larger volume in all prefrontal and hippocampal brain regions (eg, right hippocampus: β = 0.04; 95% CI, 0.02 to 0.07; *P* < .001) even when accounting for NP. The SEM model was a good fit across all cognitive domains, with prefrontal regions being associated with NP relations to language (picture vocabulary: estimate [SE], –0.03 [0.01]; *P* < .001; oral reading: estimate [SE], –0.02 [0.01]; *P* < .001), episodic memory (picture sequence: estimate [SE], –0.02 [0.01]; *P* = .008), and working memory (dimensional card sort: estimate [SE], –0.02 [0.01]; *P* = .001; flanker inhibitory control: estimate [SE], –0.01 [0.01]; *P* = .01; list sorting: estimate [SE], –0.03 [0.01]; *P* < .001) and hippocampal regions being associated with NP associations with language (picture vocabulary: estimate [SE], –0.01 [0.004]; *P* < .001) and episodic memory (picture sequence: estimate [SE], –0.01 [0.004]; *P* < 0.001).

**Conclusions and Relevance:**

In this study, NP accounted for unique variance in cognitive function and prefrontal and right hippocampal brain volume. These findings demonstrate the importance of including broader environmental influences when conceptualizing early life adversity.

## Key Points

**Question:**

What are the associations between neighborhood poverty, child cognitive performance, and brain structure after accounting for household-level poverty?

**Findings:**

This cross-sectional study of 11 875 children aged 9 and 10 years found an association between neighborhood poverty, prefrontal and hippocampal volume, and performance on cognitive tasks. These results remained even after controlling for individual household income.

**Meaning:**

The findings of this study provide evidence that the broader neighborhood context uniquely contributes to prefrontal and hippocampal development and cognitive performance and should be considered in studies of early life poverty and adversity.

## Teaser

This cross-sectional study evaluates whether neighborhood poverty is associated with cognitive function and prefrontal and hippocampal brain structure in ways that are dissociable from household socioeconomic status.

## Introduction

Early poverty has been consistently associated with cognitive function deficits and lower school and standardized test performance.^1^ Furthermore, there is evidence that developmental differences in brain maturation may mediate the association between socioeconomic status (SES) and cognitive function and educational outcomes. Much of this research has centered on household SES. Less is known about the unique association of broader neighborhood SES environments with cognitive outcomes and brain maturation in children after accounting for individual household SES. Current models indexing poverty and early life adversity do not commonly include neighborhood-level measures, despite growing evidence that consideration of the neighborhood context is important for gaining a comprehensive understanding of mechanisms associated with cognitive and educational outcomes in children. As such, the goal of the current study was to examine whether neighborhood poverty (NP) was associated with cognitive function and brain volume in regions thought to be critical for a range of cognitive functions, even after accounting for household SES.

Numerous studies have demonstrated that children from households with lower SES score significantly lower on memory, language, and cognitive control tasks,^2^ score lower on intelligence tests and tests that measure academic achievement,^3^ and are more likely to fail courses, drop out of school, and be put into special education classes.^4^ A potential pathway by which impoverished environments could influence such outcomes is via maturation of brain structures important for cognitive development. For example, the association between household poverty and reduced hippocampal volume in children has been a robust finding in the literature.^5,6,7,8,9^ There are high concentrations of glucocorticoid receptors in the hippocampus, a region that has been robustly implicated in episodic memory and memory consolidation,^10,11,12^ which makes this region vulnerable to chronic stress responses via the hypothalamic-pituitary-adrenal (HPA) axis.^13,14^ When stress is chronic, glucocorticoid release can be maladaptive, resulting in desensitization of receptors and damage to surrounding tissue.^15^ Thus, it is has been hypothesized that chronic stressors associated with household poverty may be contributing to disruptions in hippocampal development, although there are other possible pathways, such as emotional or material deprivation, disruptions in parent-child relationship, nutrition, and exposure to toxins, that may also contribute to this relation.^16,17,18,19^ Lower SES has also been implicated in impaired maturation of the prefrontal cortex, whose protracted development may make it especially vulnerable to chronically stressful environments.^1,20,21^ Chronic activation of the HPA axis might similarly affect tissue volume and region function via glucocorticoid receptors. Reduced prefrontal volume and activity have been found in individuals from lower SES households.^22^ Previous literature has demonstrated that reduced volume and activity in prefrontal regions, like the dorsolateral prefrontal cortex (DLPFC), dorsal medial PFC (DMPFC), and superior frontal gyrus (SFG), are associated with lower performance on tasks indexing cognitive function.^23,24,25,26^

Although household-level SES is clearly important in understanding child development, some research suggests that the neighborhood context may also be important, particularly in geographic locations where structural and/or explicit racism may limit neighborhoods available to individuals who belong to specific racial and minority groups regardless of their household SES (eg, redlining practices).^27^ In a longitudinal study,^28^ individuals with higher neighborhood disadvantage were at greater risk of coronary heart disease, controlling for individual SES and education. Accumulation of neighborhood disadvantage between the ages of 16 and 43 years was associated with increased allostatic load (ie, the accumulation of chronic endocrine responses to stressful life events) in adulthood, even after accounting for personal adverse living experiences.^29^ Furthermore, children living in areas with large amounts of local violence had lower vocabulary and reading scores on an IQ test, even after accounting for household SES.^30^ Similarly, children living in neighborhoods containing more academically educated professionals had higher academic achievement and higher scores on vocabulary and reading assessments. This association was not fully explained by household income and other individual family factors.^31^

Given the literature outlined above, the goals of the current study were to test the following hypotheses: (1) NP accounts for variance in cognitive performance and prefrontal and hippocampal volumes in children, even when accounting for household SES variables, and (2) NP associations with volumes of prefrontal and hippocampal regions share variance with NP associations with cognitive function in school age children, providing evidence for the plausibility of the hypothesis that brain volume mediates the association of NP with cognitive function.

## Methods

### Participants

The sample for this study consisted of baseline data from 11 875 children recruited as part of the Adolescent Brain Cognitive Development (ABCD) Study, with recruitment across 21 sites designed to mirror the demographic characteristics of the United States.^32^ Data were collected between September 2019 and October 2018, using school-based recruitment to create a sample reflecting the US population. Race and ethnicity are highly confounded with both household income and NP, as they are in the US population, reflecting ongoing structural racism. Thus, we did not include race and ethnicity as covariates in the analyses presented below. eAppendix 1 in the Supplement contains analyses showing that most key findings hold when including race/ethnicity as a covariate. Data from ABCD Release 2.0.1 were used for the current study. Informed written consent for child and parent was obtained from parents, and child participants separately completed a written assent. This work was reviewed and approved by the Washington University human participants committee. This study followed the Strengthening the Reporting of Observational Studies in Epidemiology (STROBE) reporting guideline.

Participants who had missing data on some variables were not removed from the sample data set, as multilevel models will allow estimation with missing cases as more waves of data are collected. The sample sizes used for each analysis appear in eAppendix 2 in the Supplement. Analyses using the GGally and finalfit packages indicated that missingness of key variables (household income and NP) did suggest some association with missingness of other demographic variables (eg, race/ethnicity, sex) but did not indicate that missingness of outcome variables was based on certain levels of NP and household income (eAppendix 3 in the Supplement).

### Measures

#### NP

Parents or guardians completed a residential history questionnaire, in which they provided the participant’s current home address. The participant’s primary home address was used to generate Area Deprivation Index (ADI) values,^33^ which were factor analyzed and used to create an aggregate measure of standardized NP (median, −0.22; range, −1.49 to 3.91). The final NP aggregate consisted of 9 of 17 ADI values, given that some variables with lower factor loadings seemed to reflect geographical differences in cost of living across sites that were less indicative of objective disadvantage (eg, median mortgage or rent costs) or less modern disadvantage indices (eg, percentage of homes without a telephone). Higher scores on NP indicate increased neighborhood disadvantage (eg, greater percentage of families living in poverty, increased unemployment, lower percentage of educational attainment at the neighborhood level). More information regarding calculation and distribution appears in eAppendix 4 in the Supplement; results obtained using the ADI sum score appear in eAppendix 5 in the Supplement.

#### Household SES

Household SES was measured using both household income and the Parent-Reported Financial Adversity Questionnaire (PRFQ). Household income (median, 8; range, 1-10) was the combined income of the primary caretaker and any additional household members.^34^ The PRFQ asked questions designed to determine whether families generally have enough money to pay for basic life expenses, such as food and health care.^35^ Household income and PRFQ scores were included as separate indicators of household SES because household income is a more objective measure of SES, while PRFQ indexes self-reported finances that may better account for the association of income level with area cost-of-living.

#### National Institutes of Health Toolbox Cognitive Battery

The National Institutes of Health Toolbox Cognitive Battery (NIHTB-CB) was administered to each participant.^36^ The NIHTB-CB is composed of tasks assessing 7 cognitive domains, as follows: picture vocabulary as a measure of verbal ability; flanker inhibitory control and dimensional change card sort as measures of attention and executive functioning; list sorting as a measure of working memory; pattern comparison for processing speed; picture sequencing for episodic memory; and oral reading recognition as a measure of reading ability.^36^ Scores used in the current study were age-corrected and *z* scored.

#### Imaging Procedure and Brain Segmentation

As previously described,^37,38^ participants were scanned using similar sequences on either a 3T Siemens, Phillips, or General Electric scanner with a 32-channel head coil. A 3-dimensional T1-weighted image (1-mm voxel resolution) was acquired as participants viewed the child-appropriate movie of their choice.^37^ Motion detection and correction software were used in real-time at the Siemens and GE sites.^39,40^

Based on previous literature, the a priori regions of interest were the hippocampus and 3 regions in the prefrontal cortex (ie, DLPFC, DMPFC, and SFG). FreeSurfer version 5.3.0 was used for cortical surface reconstruction and subcortical brain segmentation from the aseg atlas for hippocampal and Desikan atlas for superior frontal regions.^38^ eAppendix 6 in the Supplement describes quality control methods. DLPFC and DMPFC were parceled into genetically based subdivisions.^41^ Participant scans that were rated as unusable were not included in the released data set. As an additional follow-up to ensure that our results did not reflect T1 quality, we reran all analyses using only those children with a 0 on the artifact score (eAppendix 7 in the Supplement).

### Statistical Analysis

#### Mixed-Effects Models

Data analysis was conducted from March to June 2019. Mixed-effects models were computed using the lmer function within the lme4 package in R version 3.6.2. (R Project for Statistical Computing).^42^ Calculation of intraclass correlation coefficient revealed that site did not account for a significant amount of variance in these models (<0.01 for cognitive outcomes and approximately 0.04 for brain outcomes). A more parsimonious model that included only a random effect of family was used for the current analyses. NP and household SES variables (household income and PRFQ) were first considered in separate models (models 1 and 2) and then were included together (model 3) to assess shared vs unique variance. Age, sex, and intracranial volume (for hippocampal and prefrontal analyses) were included as covariates. All variables were standardized for ease of comparison. Estimates were chosen to optimize the restricted maximum likelihood criterion. We performed *t* tests to look at each variable using the Sattherwaithe degrees of freedom method via the lmerTest package.^43^ Multiple comparisons were corrected using false discovery rate.^44^ Statistical significance was set at *P* < .05, and all tests were 2-tailed.

#### Structural Equation Models

Causal mediation cannot be determined with cross-sectional data. Thus, we used the lavaan package in R version 3.6.3 (R Project for Statistical Computing)^45^ to conduct structural equation models (SEMs) of indirect associations to examine the plausibility of whether brain structure could mediate the association between NP and cognitive function by assessing the shared variance in NP-to-brain measures and NP-to–NIHTB-CB measures (Figure). First, an exploratory factor analysis (EFA) was performed (psych package in R) for brain regions and NIHTB-CB scores to reduce data dimensionality. The EFA suggested 2 brain factors (prefrontal and hippocampal) but indicated that the NIHTB-CB tests should each be considered individually (eAppendix 8 in the Supplement). Second, indirect-effects models were created, which set NP, prefrontal, and hippocampal factors as free to vary and included household income, age, sex, and intracranial volume as covariates. Fit indices (comparative fit index [CFI], root mean square error of approximation [RMSEA], and standardized root mean square residual [SRMR]) assessed whether the indirect-effects model fit the data well. Models were confirmed using a subset of the data that included only 1 child per family (ie, children with no siblings; n = 9988) to rule out family dependency confounds.

**Figure.**
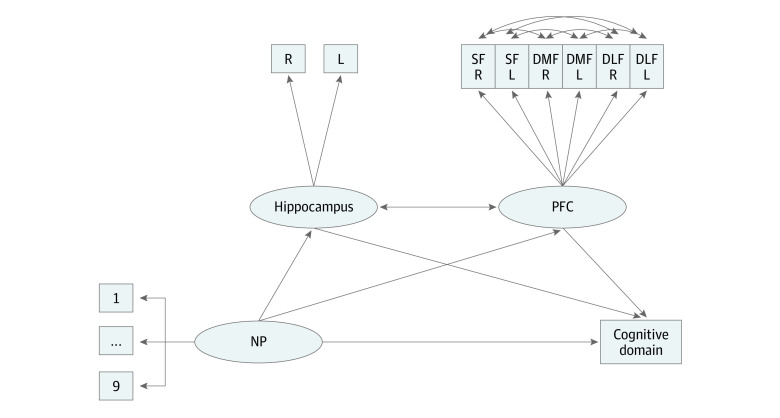
Schematic of SEM With Neighborhood Poverty and Brain Region Associations With Cognitive Performance DLF indicates dorsolateral prefrontal cortex; DMF, dorsomedial prefrontal cortex; L, left; NP, neighborhood poverty; R, right; SF, superior frontal gyrus.

## Results

In this sample of 11 875 children, 5678 (47.8%) were girls. All participants were aged 9 or 10 years. Table 1 presents sex and race/ethnicity proportions. Household income and PRFQ were negatively correlated (*r* = −0.42; *P* < .001). NP was negatively correlated with household income (*r* = −0.56; *P* < .001) and positively correlated with PRFQ (*r* = 0.27; *P* < .001).

**Table 1.** Demographic Percentages in Current Sample

Demographic variable	Children, No. (%) (N = 11 875)
Sex	
Female	5678 (47.8)
Male	6184 (52.1)
NA	13 (0.1)
Race^a^	
White	8803 (74.1)
Black	2515 (21.2)
Asian	822 (6.9)
Native American or Alaskan	411 (3.5)
Native Hawaiian or Pacific Islander	37 (0.3)
Other race	799 (6.7)
Did not know or did not disclose	163 (1.3)
Ethnicity	
Hispanic or Latinx	2407 (20.3)

^a^ Counts for race exceed total sample count (n = 11 875) because parents were permitted to endorse multiple racial categories to describe child’s racial identity.

### Association of NP With NIHTB-CB Scores

eAppendix 9 in the Supplement presents results using unstandardized variables. As shown in Table 2, without NP in the models (model 1), higher household income was associated with higher scores across all measures of the NIHTB-CB (eg, total composite: β = 0.38; 95% CI, 0.36 to 0.40; *P* < .001). Lower PRFQ was associated with higher scores on picture vocabulary, oral reading, dimensional card sort, and picture sequencing tasks. Without household income or PRFQ in the models (model 2), greater NP was also significantly associated with lower scores across all measures of the NIHTB-CB (eg, total composite: β = −0.41; 95% CI, −0.44 to −0.39; *P* < .001). Importantly, when household income, PRFQ, and NP were in the same model (model 3), both NP and household income continued to be independently associated with each of the scores (total composite, NP: β = −0.18; 95% CI, −0.21 to −0.15; *P* < .001; total composite, household income: β = 0.30; 95% CI, 0.28 to 0.33; *P* < .001), although PRFQ did not. Examination of unstandardized variables (eAppendix 9 in the Supplement) indicated that for every unit increase in NP, children scored 3.22 points lower on the NIHTB-CB composite, even when accounting for household SES.

**Table 2.** Model Output for NIHTB-CB Measures

NIHTB-CB measures	Neighborhood poverty			Household income			PRFQ		
	β (95% CIs)	*t*	*P* value	β (95% CIs)	*t*	*P* value	β (95% CIs)	*t*	*P* value
Model 1^a^									
Picture vocabulary	NA	NA	NA	0.36 (0.34 to 0.38)	34.82	<.001	–0.04 (–0.06 to –0.02)	–2.63	.01
Oral reading	NA	NA	NA	0.28 (0.26 to 0.3)	25.89	<.001	–0.04 (–0.06 to –0.02)	–3.52	<.001
Dimensional card sort	NA	NA	NA	0.18 (0.16 to 0.2)	16.7	<.001	–0.03 (–0.06 to –0.01)	–3.08	<.01
Flanker inhibitory control	NA	NA	NA	0.16 (0.14 to 0.19)	15.18	<.001	0.00 (–0.02 to 0.02)	–0.2	.84
List sorting	NA	NA	NA	0.28 (0.26 to 0.31)	26.73	<.001	–0.02 (–0.04 to 0)	–1.62	.11
Pattern comparison	NA	NA	NA	0.11 (0.09 to 0.13)	9.93	<.001	–0.02 (–0.04 to 0)	–1.95	.05
Picture sequencing	NA	NA	NA	0.18 (0.16 to 0.2)	16.75	<.001	–0.03 (–0.05 to –0.01)	–2.76	.01
Total composite	NA	NA	NA	0.38 (0.36 to 0.4)	36.91	<.001	–0.05 (–0.07 to –0.03)	–4.35	<.001
Model 2^b^									
Picture vocabulary	–0.4 (–0.42 to –0.38)	–35.85	<.001	NA	NA	NA	NA	NA	NA
Oral reading	–0.27 (–0.29 to –0.24)	–23.3	<.001	NA	NA	NA	NA	NA	NA
Dimensional card sort	–0.22 (–0.25 to –0.2)	–19.65	<.001	NA	NA	NA	NA	NA	NA
Flanker inhibitory control	–0.19 (–0.21 to –0.16)	–16.26	<.001	NA	NA	NA	NA	NA	NA
List sorting	–0.3 (–0.32 to –0.27)	–26.11	<.001	NA	NA	NA	NA	NA	NA
Pattern comparison	–0.14 (–0.17 to –0.12)	–12.71	<.001	NA	NA	NA	NA	NA	NA
Picture sequencing	–0.19 (–0.21 to –0.17)	–16.66	<.001	NA	NA	NA	NA	NA	NA
Total composite	–0.41 (–0.44 to –0.39)	–37.05	<.001	NA	NA	NA	NA	NA	NA
Model 3^c^									
Picture vocabulary	–0.18 (–0.21 to –0.15)	–12.43	<.001	0.28 (0.25 to 0.3)	21.98	<.001	–0.04 (–0.06 to –0.02)	–3.63	.11
Oral reading	–0.07 (–0.1 to –0.04)	–4.58	<.001	0.25 (0.22 to 0.27)	18.72	<.001	–0.04 (–0.06 to –0.02)	–3.44	.11
Dimensional card sort	–0.12 (–0.15 to –0.09)	–7.72	<.001	0.13 (0.1 to 0.15)	9.63	<.001	–0.03 (–0.05 to 0.01)	–2.76	.21
Flanker inhibitory control	–0.1 (–0.13 to –0.07)	–6.32	<.001	0.12 (0.1 to 0.15)	9.19	<.001	NA	NA	NA
List sorting	–0.13 (–0.16 to –0.1)	–8.62	<.001	0.23 (0.2 to 0.25)	17.4	<.001	NA	NA	NA
Pattern comparison	–0.09 (–0.12 to –0.06)	–6.16	<.001	0.07 (0.04 to 0.09)	5.03	<.001	NA	NA	NA
Picture sequencing	–0.07 (–0.1 to –0.04)	–4.84	<.001	0.14 (0.12 to 0.17)	10.8	<.001	–0.03 (–0.05 to –0.01)	–2.36	.12
Total composite	–0.18 (–0.21 to –0.15)	–12.44	<.001	0.3 (0.28 to 0.33)	23.92	<.001	–0.04 (–0.06 to –0.2)	–3.71	.12

Abbreviation: NA, not applicable; NIHTB-CB, National Institutes of Health Toolbox–Cognitive Measures; PRFQ, Parent-Reported Financial Adversity Questionnaire.

^a^ Model 1 included household income and PRFQ as factors with age and sex included as covariates.

^b^ Model 2 included neighborhood poverty as a factor with age and sex included as covariates.

^c^ Model 3 included household income, PRFQ, and neighborhood poverty as factors with age and sex included as covariates.

### Association of NP With Hippocampal Volume

eAppendix 9 in the Supplement presents results using unstandardized variables. As shown in Table 3, when NP was not in the models (model 1), higher household income was significantly associated with increased volume in bilateral hippocampus (eg, right hippocampus: β = 0.06; 95% CI, 0.05 to 0.08; *P* < .001). Lower PRFQ scores were significantly associated with increased volume in right hippocampus (β = –0.02; 95% CI, –0.04 to –0.01; *P* = .01). In model 2, higher NP was associated with decreased volume in both right and left hippocampus (right: β = –0.08; 95% CI, –0.10 to –0.06; *P* < .001; left: β = –0.06; 95% CI, –0.08 to –0.05; *P* = .04). With both household SES variables and NP included (model 3), both NP and household income continued to be independently associated with right hippocampal volume (NP: β = –0.04; 95% CI, –0.06 to –0.01; *P* = .01; household income: β = 0.04; 95% CI, 0.02 to 0.07; *P* < .001), while PRFQ was not. For left hippocampal volume, household income was significantly associated (β = 0.06; 95% CI, 0.04 to 0.08; *P* < .001), but NP was not (β = –0.02; 95% CI, –0.04 to –0.01; *P* = .21).

**Table 3.** Model Output for Brain Regions of Interest

Brain ROIs	Neighborhood poverty			Household income			PRFQ		
	β (95% CIs)	*t*	*P* value	β (95% CIs)	*t*	*P* value	β (95% CIs)	*t*	*P* value
Model 1^a^									
Right hippocampus	NA	NA	NA	0.06 (0.05 to 0.08)	7.16	<.001	–0.02 (–0.04 to –0.01)	–2.63	.01
Left hippocampus	NA	NA	NA	0.07 (0.05 to 0.09)	7.73	<.001	–0.01 (–0.03 to 0.01)	–1.32	.19
Right SFG	NA	NA	NA	0.10 (0.08 to 0.12)	11.35	<.001	–0.02 (–0.03 to 0)	–1.78	.09
Left SFG	NA	NA	NA	0.09 (0.07 to 0.10)	10.22	<.001	–0.01 (–0.03 to 0)	–1.73	.09
Right DLPFC	NA	NA	NA	0.12 (0.10 to 0.13)	14.58	<.001	–0.02 (–0.03 to 0)	–2.35	.03
Left DLPFC	NA	NA	NA	0.11 (0.10 to 0.13)	14.39	<.001	–0.01 (–0.03 to 0)	–1.93	.07
Right DMPFC	NA	NA	NA	0.10 (0.08 to 0.12)	12.56	<.001	–0.02 (–0.04 to –0.01)	–2.68	.01
Left DMPFC	NA	NA	NA	0.10 (0.08 to 0.11)	12.25	<.001	–0.02 (–0.03 to 0)	–2.5	.01
Model 2^b^									
Right hippocampus	–0.08 (–0.10 to –0.06)	–8.35	<.001	NA	NA	NA	NA	NA	NA
Left hippocampus	–0.06 (–0.08 to –0.05)	–6.77	.04	NA	NA	NA	NA	NA	NA
Right SFG	–0.11 (–0.12 to –0.09)	–11.5	<.001	NA	NA	NA	NA	NA	NA
Left SFG	–0.10 (–0.12 to –0.08)	–10.97	<.001	NA	NA	NA	NA	NA	NA
Right DLPFC	–0.15 (–0.17 to –0.13)	–17.5	<.001	NA	NA	NA	NA	NA	NA
Left DLPFC	–0.13 (–0.15 to –0.12)	–16.75	<.001	NA	NA	NA	NA	NA	NA
Right DMPFC	–0.12 (–0.13 to –0.1)	–14.21	<.001	NA	NA	NA	NA	NA	NA
Left DMPFC	–0.11 (–0.12 to –0.09)	–13.98	<.001	NA	NA	NA	NA	NA	NA
Model 3^c^									
Right hippocampus	–0.04 (–0.06 to –0.01)	–2.89	.01	0.04 (0.02 to 0.07)	4.14	<.001	–0.02 (–0.04 to –0.01)	–2.6	.05
Left hippocampus	–0.02 (–0.04 to –0.01)	–1.29	.21	0.06 (0.04 to 0.08)	5.55	<.001	NA	NA	NA
Right SFG	–0.05 (–0.08 to –0.03)	–4.24	<.001	0.08 (0.06 to 0.1)	7.4	<.001	NA	NA	NA
Left SFG	–0.05 (–0.07 to –0.03)	–4.29	<.001	0.07 (0.05 to 0.09)	6.43	<.001	NA	NA	NA
Right DLPFC	–0.09 (–0.12 to –0.07)	–8.43	<.001	0.08 (0.06 to 0.1)	7.83	<.001	–0.01 (–0.03 to 0)	–1.68	.09
Left DLPFC	–0.08 (–0.1 to –0.06)	–7.7	<.001	0.07 (0.06 to 0.09)	8.06	<.001	NA	NA	NA
Right DMPFC	–0.07 (–0.09 to –0.05)	–6.08	<.001	0.07 (0.05 to 0.09)	7.54	<.001	–0.01 (–0.03 to 0)	–1.72	.12
Left DMPFC	–0.06 (–0.08 to –0.04)	–5.88	<.001	0.07 (0.05 to 0.08)	7.25	<.001	–0.01 (–0.03 to 0)	–1.69	.12

Abbreviations: DLPFC, dorsal lateral prefrontal cortex; DMPFC, dorsal medial prefrontal cortex; NA, not applicable; PRFQ, Parent-Reported Financial Adversity Questionnaire; ROI, region of interest; SFG, superior frontal gyrus.

^a^ Model 1 included household income and PRFQ as factors with age, sex, and intracranial volume included as covariates.

^b^ Model 2 included neighborhood poverty as a factor, with age, sex, and intracranial volume included as covariates.

^c^ Model 3 included household income, PRFQ, and neighborhood poverty as factors with age, sex, and intracranial volume included as covariates.

### Associations With Prefrontal Volumes

As shown in Table 3, when considered alone (model 1), greater household income was associated with increased volume in right and left SFG (right: β = 0.10; 95% CI, 0.08 to 0.12; *P* < .001; left: β = 0.09; 95% CI, 0.07 to 0.10; *P* < .001), DLPFC (eg, right: β = 0.12; 95% CI, 0.10 to 0.13; *P* < .001) and DMPFC (eg, right: β = 0.10; 95% CI, 0.08 to 0.12; *P* < .001). Lower PRFQ was also associated with increased volume in right DLPFC and both right and left DMPFC. When considered alone (model 2), higher NP was associated with decreased volume in both right and left SFG (right: β = –0.11; 95% CI, –0.12 to –0.09; *P* < .001; left: β = –0.10; 95% CI, –0.12 to –0.08; *P* < .001), DLPFC (eg, right: β = –0.15; 95% CI, –0.17 to –0.13; *P* < .001), and DMPFC (eg, right: β = –0.12; 95% CI, –0.13 to –0.10; *P* < .001. When included together, NP and household income were each significantly independently associated with volume in right and left hemispheres of each of the prefrontal regions. Greater NP was associated with volume in the DLPFC (eg, right DLPFC: β = −0.09; 95% CI, −0.12 to −0.07; *P* < .001), DMPFC (eg, right DMPC: β = −0.07; 95% CI, −0.09 to −0.05; *P* < .001), and SFG (eg, right SFG: β = −0.05; 95% CI, −0.08 to −0.03; *P* < .001). Follow-up exploratory analyses were conducted to determine whether NP’s association with brain volume extended to other prefrontal regions.

### SEM Analyses

For each cognitive task, the model (which included both prefrontal and hippocampal regions simultaneously) was supported as a good fit for the databased on CFI, RMSEA, and SRMR indices (Table 4). Both the prefrontal and hippocampal factors were significantly associated with NP associations with picture vocabulary (prefrontal: estimate [SE], –0.03 [0.01]; *P* < .001; hippocampal: estimate [SE], –0.01 [0.004]; *P* < .001), oral reading (prefrontal: estimate [SE], –0.02 [0.01]; *P* < .001; hippocampal: estimate [SE], –0.01 [0.004]; *P* < .001), and picture sequence (prefrontal: estimate [SE], –0.01 [0.004]; *P* = .008; hippocampal: estimate [SE], –0.01 [0.004]; *P* < 0.01) tasks (Table 4). However, only the prefrontal factor was significantly associated with NP associations with the dimensional card sort (estimate [SE], –0.02 [0.01]; *P* = .001), flanker inhibitory control (estimate [SE], –0.01 [0.01]; *P* = .01), and list sorting (estimate [SE], –0.03 [0.01]; *P* < .001) tasks.

**Table 4.** Model Output for SEM Analyses Investigating Plausibility of Mediation^a^

Factor	Path a, NP and brain factors			Path b, brain factors and cognitive tasks			Path a × b*,* indirect effect			Path c, NP and cognitive tasks with brain factors in model		
	Estimate (SE)	*z*	*P* value	Estimate (SE)	*z*	*P* value	Estimate (SE)	*z*	*P* value	Estimate (SE)	*z*	*P* value
**Picture vocabulary**												
NP	NA	NA	NA	NA	NA	NA	NA	NA	NA	–0.15 (0.02)	–10.26	<.001
Prefrontal factor	–0.23 (0.01)	–21.27	<.001	0.15 (0.02)	7.41	<.001	–0.03 (0.01)	–7.02	<.001	NA	NA	NA
Hippocampal factor	–0.19 (0.01)	–16.15	<.001	0.07 (0.02)	3.87	<.001	–0.01 (0.004)	–3.77	<.001	NA	NA	NA
**Oral reading**												
NP	NA	NA	NA	NA	NA	NA	NA	NA	NA	–0.06 (0.02)	–3.61	<.001
Prefrontal Factor	–0.23 (0.01)	–21.27	<.001	0.08 (0.02)	3.82	<.001	–0.02 (0.01)	–3.75	<.001	NA	NA	NA
Hippocampal factor	–0.19 (0.01)	–16.15	<.001	0.07 (0.02)	3.68	<.001	–0.01 (0.004)	–3.52	<.001	NA	NA	NA
**Dimensional card sort**												
NP	NA	NA	NA	NA	NA	NA	NA	NA		–0.1 (0.02)	–6.75	<.001
Prefrontal factor	–0.23 (0.01)	–21.27	<.001	0.07 (0.02)	3.33	<.01	–0.02 (0.01)	–3.27	.001	NA	NA	NA
Hippocampal factor	–0.19 (0.01)	–16.15	<.001	0.04 (0.02)	1.9	.06	–0.01 (0.004)	–1.89	.06	NA	NA	NA
**Flanker inhibitory control **												
NP	NA	NA	NA	NA	NA	NA	NA	NA	NA	–0.08 (0.02)	–4.63	<.001
Prefrontal factor	–0.23 (0.01)	–21.27	<.001	0.06 (0.02)	2.64	.01	–0.01 (0.01)	–2.62	.01	NA	NA	NA
Hippocampal factor	–0.19 (0.01)	–16.15	<.001	0.04 (0.02)	1.85	.06	–0.01 (0.004)	–1.84	.07	NA	NA	NA
**List sorting**												
NP	NA	NA	NA	NA	NA	NA	NA	NA	NA	–0.12 (0.02)	–7.63	<.001
Prefrontal factor	–0.23 (0.01)	–21.27	<.001	0.12 (0.02)	5.73	<.001	–0.03 (0.01)	–5.56	<.001	NA	NA	NA
Hippocampal factor	–0.19 (0.01)	–16.15	<.001	0.02 (0.02)	0.92	.36	–0.01 (0.004)	–0.92	.36	NA	NA	NA
**Pattern comparison**												
NP	NA	NA	NA	NA	NA	NA	NA	NA	NA	–0.07 (0.02)	–4.5	<.001
Prefrontal factor	–0.23 (0.01)	–21.27	<.001	0.04 (0.02)	1.83	.07	–0.01 (0.01)	–1.82	.07	NA	NA	NA
Hippocampal factor	–0.19 (0.01)	–16.15	<.001	0.02 (0.02)	1.16	.25	–0.01 (0.004)	–1.15	.25	NA	NA	NA
**Picture sequence**												
NP	NA	NA	NA	NA	NA	NA	NA	NA	NA	–0.07 (0.02)	–4.32	<.001
Prefrontal factor	–0.23 (0.01)	–21.27	<.001	0.06 (0.02)	2.97	.008	–0.02 (0.01)	–2.93	<.001	NA	NA	NA
Hippocampal factor	–0.19 (0.01)	–16.15	<.001	0.07 (0.02)	3.49	<.001	–0.01 (0.004)	–3.42	<.001	NA	NA	NA

Abbreviations: NA, not applicable; NP, neighborhood poverty.

^a^ Covariates included: age, sex, household income, and intracranial volume. Fit indices suggest that models were a good fit for the data: Prefrontal models (CFI = 0.936, RMSEA = 0.087, SRMR = 0.071); Hippocampal models (CFI = 0.940, RMSEA = 0.084, SRMR = 0.053). NP was modeled as the predictor with prefrontal and hippocampal factors as mediators, with separate cognitive tasks as the outcome (7 models total).

## Discussion

Consistent with our hypothesis, the results of this study indicated that NP was significantly associated with a range of cognitive function domains as well as bilateral prefrontal and right hippocampal volumes, even after accounting for individual household income and PRFQ. Furthermore, the SEMs provided evidence that it is plausible that variation in prefrontal and hippocampal brain volume may mediate the association between NP and cognitive outcomes, with the relative contributions of prefrontal vs hippocampal volumes varying across cognitive domains. The effect sizes for associations with household SES were generally stronger than those for NP; therefore, we are not arguing that NP is more important than household SES. However, the fact that NP accounted for variance in addition to household SES supports the idea that consideration of the neighborhood context is important in conceptualizing the complex environment of the developing brain.

Higher levels of NP were independently and significantly associated with lower scores on all cognitive domains. Standardized effect sizes were conventionally small but nonetheless meaningful in indicating the need to assess the ways in which neighborhood characteristics may contribute to brain structure and cognitive development, as even small differences could build over time into larger differences in functional outcomes. These findings are consistent with previous literature demonstrating poorer school and cognitive performance among children raised in impoverished environments^1,3,4,46^ and provide further support for the importance of consideration of the neighborhood context in understanding child outcomes. A key question is what aspects of NP are critical for explaining cognitive function among children that are not fully accounted for by individual household function. A possibility is that the neighborhood context might be associated with school environment and/or funding, and the development of a range of cognitive processes are likely sensitive to school context and resources. Future research should include indices of local school context to investigate this hypothesis further. Importantly, the size and nature of the sample (mirroring the US child population) suggest that these findings are likely to be generalizable to broader normative populations.

Consistent with prior literature,^6,7,8,9,10,11,12^ higher household income was associated with increased volumes in all hippocampal and prefrontal brain regions. Additionally, higher NP was significantly and independently associated with reduced brain volume in DLPFC, DMPFC, SFG, and right hippocampal regions. Interestingly, the effect sizes for both NP and household SES variables’ association with brain volume were smaller than those for the cognitive measures. It is possible that the bigger effect sizes for cognitive function reflect a strong association between NP and school funding and quality, which may have a stronger association with cognitive function than brain metrics. In contrast, our NP measure may not be as strongly associated with other potential correlates, which may be more directly related to brain development. For example, individuals living in poverty are more likely to be exposed to lead and air pollutants in childhood.^47,48,49^ As such, it will be important to look at lead (and other toxin) exposure to determine whether it might be associated with brain volume in ways not captured by the current measure of NP. Additionally, NP was not independently associated with orbitofrontal cortex volume, suggesting that some specificity in NP relations to prefrontal regions (eAppendix 10 in the Supplement).

The SEM results provided evidence that prefrontal and hippocampal brain volumes explained variance in the association between NP and cognitive task performance, even when controlling for household income. Interestingly, the relative associations of prefrontal cortex and hippocampal volumes varied across cognitive measures. Both prefrontal and hippocampal volumes were significantly associated with the associations between NP and tasks indexing language ability and crystallized intelligence.^50,51^ The hippocampus is important for the consolidation of long-term information, and the role of the prefrontal cortex in language processing and production has been well-established.^52,53,54^ Similarly, both prefrontal and hippocampal volumes were significantly associated with the association between NP and episodic memory. This is consistent with a large body of literature identifying the hippocampus as critical for consolidation of episodic memories with prefrontal regions providing organizational support for encoding.^11,12,55^ In contrast, only prefrontal volumes were significantly associated with the association between NP and executive function and working memory tasks. This finding is consistent with literature suggesting that prefrontal regions support the top-down processing of stimuli, allowing for flexible and nonautomatic behavioral responses.^56^ These results provide evidence supporting the plausibility of a mediation, which can be assessed in subsequent studies as additional waves of data are collected.

### Limitations

This study has limitations. All of the neighborhood variables were focused on SES. The inclusion of other neighborhood characteristics that may not directly index SES (physical or social factors such as number of grocery stores, green space, amount of litter, air pollution)^47,48,49^ might further elucidate associations between environmental context and various outcomes.^57^ Another limitation is that the current data set is cross-sectional, which means that direction of association cannot be determined. As additional waves of ABCD data accrue, the models tested here should be extended longitudinally to make a more compelling case for mediation and direction of effect.

## Conclusions

This study found evidence for independent associations of household and neighborhood environment with brain and cognitive outcomes in preadolescent children. The study also provided evidence consistent with a pathway wherein variation in prefrontal and hippocampal volume partially explains the association between NP and scores on cognitive tests. The differential prefrontal and hippocampal associations are consistent with what would be expected for the different cognitive domains. These results provide evidence that the inclusion of neighborhood variables is a warranted addition in models of how early lived environments are associated with brain maturation and cognitive outcomes, which may inform the types of interventions offered to children from disadvantaged backgrounds.

## Notes

Supplement. **eAppendix 1. **Distributions of NP and Household Income in Black vs White Participants and Model Results Including Race/Ethnicity as a Covariate **eAppendix 2. **Sample Sizes Used for Each MLM Analysis **eAppendix 3. **Missingness Within the Study Sample and Comparison of Missing Values Among Demographic and Outcome Variables **eAppendix 4. **Distribution of NP and Household Income in Overall Sample and Additional Information on Measure of NP, PRFQ, and Household Income **eAppendix 5. **MLM Results Tables With ADI Weighted Sum as Independent Variable **eAppendix 6. **FreeSurfer QC Methods **eAppendix 7. **ML Model 3 Results Using Only Those Participants With a 0 on the Artifact Score **eAppendix 8. **NIH Toolbox-CB and Brain ROI EFA **eAppendix 9. **MLM Unstandardized Results Tables **eAppendix 10. **MLM Results Looking at Orbital Frontal Regions as the Outcome Variable
