# Supplementary material for: Assessment of Neighborhood Poverty, Cognitive Function, and Prefrontal and Hippocampal Volumes in Children
Source: JAMA Netw Open. 2020 Nov 3;3(11):e2023774. doi: 10.1001/jamanetworkopen.2020.23774 (PMC7610187; doi:10.1001/jamanetworkopen.2020.23774)
Supplement: Supplement. — eAppendix 1. Distributions of NP and Household Income in Black vs White Participants and Model Results Including Race/Ethnicity as a Covariate eAppendix 2. Sample Sizes Used for Each MLM Analysis eAppendix 3. Missingness Within the Study Sample and Comparison of Missing Values Among Demographic and Outcome Variables eAppendix 4. Distribution of NP and Household Income in Overall Sample and Additional Information on Measure of NP, PRFQ, and Household Income eAppendix 5. MLM Results Tables With ADI Weighted Sum as Independent Variable eAppendix 6. FreeSurfer QC Methods eAppendix 7. ML Model 3 Results Using Only Those Participants With a 0 on the Artifact Score eAppendix 8. NIH Toolbox-CB and Brain ROI EFA eAppendix 9. MLM Unstandardized Results Tables eAppendix 10. MLM Results Looking at Orbital Frontal Regions as the Outcome Variable [file jamanetwopen-e2023774-s001.pdf]

Supplemental Online Content

Taylor RL, Cooper SR, Jackson JJ, Barch DM. Assessment of neighborhood poverty, cognitive function, and prefrontal and hippocampal volumes in children. *JAMA Netw Open*. 2020;3(11):e2023774. doi:10.1001/jamanetworkopen.2020.23774

eAppendix 1. Distributions of NP and Household Income in Black vs White Participants and Model Results Including Race/Ethnicity as a Covariate

eAppendix 2. Sample Sizes Used for Each MLM Analysis

eAppendix 3. Missingness Within the Study Sample and Comparison of Missing Values Among Demographic and Outcome Variables

eAppendix 4. Distribution of NP and Household Income in Overall Sample and Additional Information on Measure of NP, PRFQ, and Household Income

eAppendix 5. MLM Results Tables With ADI Weighted Sum as Independent Variable

eAppendix 6. FreeSurfer QC Methods

eAppendix 7. ML Model 3 Results Using Only Those Participants With a 0 on the Artifact Score

eAppendix 8. NIH Toolbox-CB and Brain ROI EFA

eAppendix 9. MLM Unstandardized Results Tables

eAppendix 10. MLM Results Looking at Orbital Frontal Regions as the Outcome Variable

This supplemental material has been provided by the authors to give readers additional information about their work.

Supplemental Methods and Results

(a) Distributions of NP and household income in Black vs. White participants and model results including race/ethnicity as a covariate
(b) Sample sizes used for each the MLM analyses
(c) Missingness within the study sample and comparison of missing values among demographic and outcome variables

(d) Distribution of NP and household income in overall sample and additional information on measure of NP, PRFQ, and household income
(e) MLM results tables with ADI weighted sum as independent variable

(f) FreeSurfer QC methods

(g) ML Model 3 results using only those participants with a 0 on the artifact score

(h) NIH Toolbox-CB and Brain ROI EFA

(i) MLM unstandardized results tables

(j) MLM results looking at orbital frontal regions as the outcome variable

© 2020 Taylor RL et al. *JAMA Network Open.*

eAppendix 1. Distributions of NP and Household Income in Black vs White Participants and Model Results Including Race/Ethnicity as a Covariate

The ABCD sampling was designed to mirror the US population. As such, race and ethnicity are highly confounded with both household income and neighborhood poverty in the ABCD sample, as they are in the US population, reflecting ongoing structural and explicit racism. As shown in eFigures 1 and 2, the distribution of both household income and neighborhood poverty are very different among black and white youth in the ABCD sample, as they are in the US. Thus, we did not include race and ethnicity as covariates in the analyses in the main text. At the request of reviewers, we conducted additional analyses with race/ethnicity as a covariate. As shown in eTables 1 and 2, all of the relations of household income and neighborhood SES in Models 1 and 2 (household and neighborhood examined separately) held when including race/ethnicity as a covariate. For the cognitive measures, as shown in eTable 1, in Model 3 (household and neighborhood SES examined simultaneously), all of the relations to household income held with race/ethnicity as a covariate, and all of the relations of neighborhood SES also held other than for Picture Sequence (a measure of episodic memory). For the brain volume measures, as shown in eTable 2, in Model 3 (household and neighborhood SES examined simultaneously), all of the relations to household income held with race/ethnicity as a covariate, and the relations of neighborhood SES to both right and left DLPFC volume held (the right hippocampus and bilateral SFG and DMPFC did not). For the structural equation models investigating evidence to support future mediation (eTable 3), all of the same results were significant other than the prefrontal factor in association with Picture Sequencing.

© 2020 Taylor RL et al. *JAMA Network Open.*

eFigure 1. Distribution of Neighborhood Poverty in Black vs. White Participants

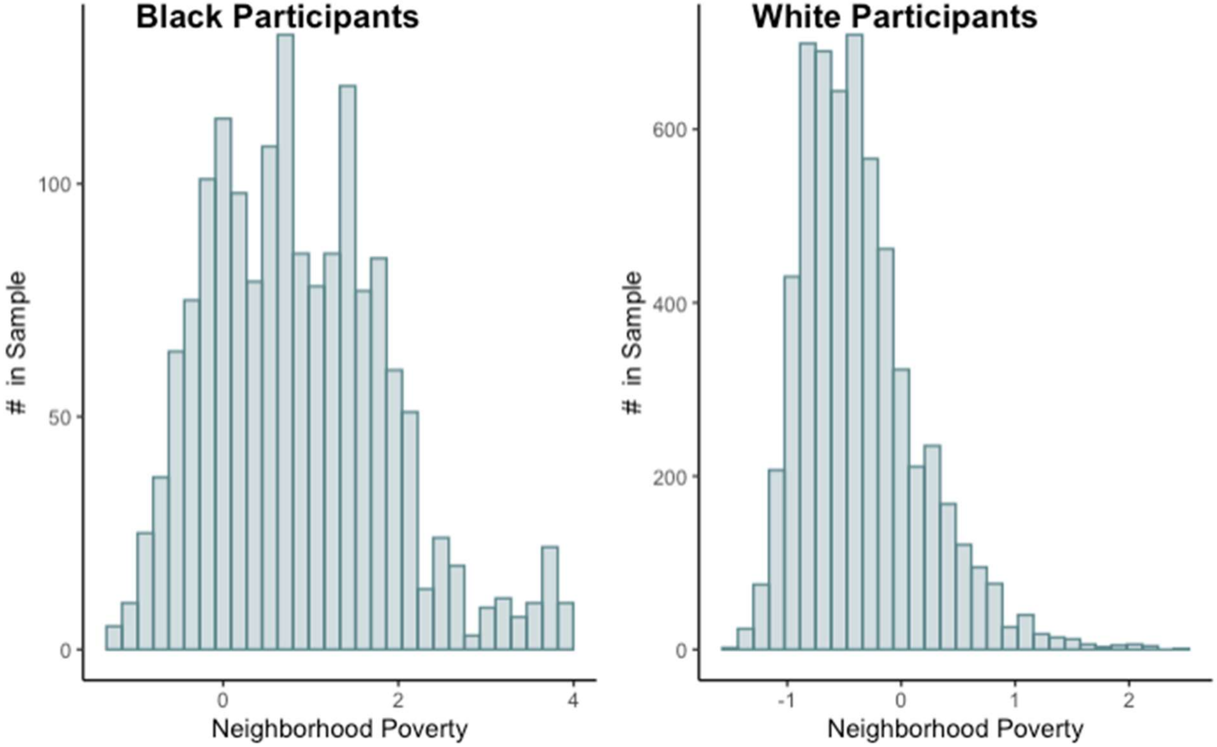

Mdn = 0.76<br>Range = 5.15 (min = -1.25, max = 3.9)	*Mdn* = -0.44<br>Range = 3.96 (min=-1.49, max = 2.47)

© 2020 Taylor RL et al. *JAMA Network Open.*

eFigure 2. Distribution of Household Income in Black vs. White Participants

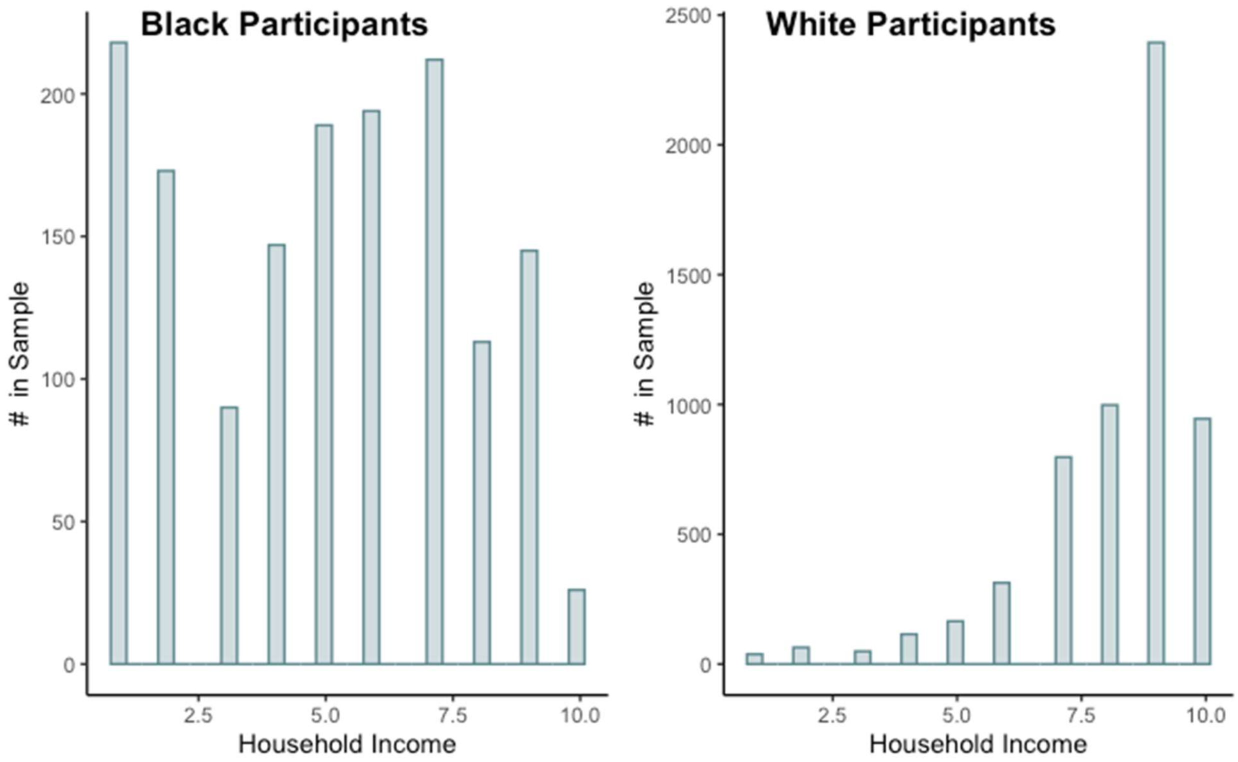

Mdn = 5
Range = 9 (min = 1, max = 10)

Mdn = 9
Range = 9 (min = 1, max = 10)

© 2020 Taylor RL et al. *JAMA Network Open.*

eTable 1. Model Output for NIH Toolbox-CB Measures with Race/Ethnicity as a Covariate

	Neighborhood Poverty				Household Income				PRFQ			
**NIH Toolbox-CB Measures**	***Std. b***	**CIs**	***t***	***p***	***Std. b***	**CIs**	***t***	***p***	***Std. b***	**CIs**	***t***	***p***
**Model 1[a]**												
Picture Vocab.					0.25	0.23 to 0.27	22.15	<0.001	-0.03	-0.05 to -0.01	-3.2	<0.01
Oral Reading					0.24	0.21 to 0.26	19.82	<0.001	-0.03	-0.05 to -0.01	0.8	0.42
Dim. Card Sort					0.13	0.11 to 0.15	10.91	<0.001	-0.03	-0.05 to -0.01	-2.49	0.01
Flanker					0.14	0.12 to 0.17	11.87	<0.001	0	-0.02 to 0.02	0.09	0.93
List Sorting					0.22	0.2 to 0.24	18.66	<0.001	-0.01	-0.03 to 0.01	-0.85	0.4
Pattern Comp.					0.07	0.05 to 0.1	5.97	<0.001	-0.02	-0.04 to 0.01	-1.49	0.14
Picture Seq.					0.12	0.1 to 0.15	10.28	<0.001	-0.02	-0.04 to 0	-1.78	0.07
Total Composite					0.29	0.27 to 0.31	25.51	<0.001	-0.03	-0.05 to -0.01	-3.37	<0.01
**Model 2[b]**												
Picture Vocab.	-0.22	-0.24 to -0.2	-17.48	<0.001								
Oral Reading	-0.18	-0.2 to -0.15	-13.17	<0.001								
Dim. Card Sort	-0.15	-0.17 to -0.12	-11.06	<0.001								
Flanker	-0.15	-0.18 to -0.12	-11.25	<0.001								
List Sorting	-0.18	-0.21 to -0.16	-14	<0.001								
Pattern Comp.	-0.1	-0.12 to -0.07	-7.36	<0.001								
Picture Seq.	-0.09	-0.12 to -0.07	-6.94	<0.001								
Total Composite	-0.26	-0.28 to -0.23	-20.16	<0.001								
**Model 3[c]**												
Picture Vocab.	-0.08	-0.11 to -0.05	-5.56	<0.001	0.22	0.19 to 0.24	17.13	<0.001	-0.03	-0.05 to -0.01	-3.16	<0.01
Oral Reading	-0.03	-0.06 to -0.01	-2.02	0.04	0.22	0.2 to 0.25	16.73	<0.001				
Dim. Card Sort	-0.08	-0.11 to -0.05	-4.94	<0.001	0.11	0.08 to 0.13	7.75	<0.001	-0.03	-0.05 to -0.01	-2.48	0.01
Flanker	-0.08	-0.12 to -0.05	-5.21	<0.001	0.12	0.09 to 0.14	8.56	<0.001				
List Sorting	-0.08	-0.12 to -0.05	-4.87	<0.001	0.19	0.17 to 0.22	14.66	<0.001				
Pattern Comp.	-0.07	-0.1 to -0.04	-4.22	<0.001	0.05	0.03 to 0.08	3.88	<0.001				
Picture Seq.	-0.02	-0.05 to 0.01	-1.09	0.27	0.11	0.09 to 0.14	8.3	<0.001				
Total Composite	-0.1	-0.13 to -0.07	-6.74	<0.001	0.25	0.23 to 0.28	19.98	<0.001	-0.03	-0.05 to -0.01	-3.23	<0.01

Abbreviation: *Std. b* = standardized coefficient estimates; PRFQ = Parent-Reported Financial Adversity Questionnaire

[a] Model 1 included household income and PRFQ as predictors with age, sex, and race/ethnicity included as covariates.
[b] Model 2 included NP as a predictor with age, sex, and race/ethnicity included as covariates.
[c] Model 3 included household income, PRFQ, and NP as predictors with age, sex, and race/ethnicity included as covariates.

© 2020 Taylor RL et al. *JAMA Network Open.*

eTable 2. Model Output for Brain Regions of Interest (ROIs) with Race/Ethnicity as a Covariate

	Neighborhood Poverty				Household Income				PRFQ			
**Brain ROIs**	***Std. b***	**CIs**	***t***	***p***	***Std. b***	**CIs**	***t***	***p***	***Std. b***	**CIs**	***t***	***p***
**Model 1[a]**												
R Hippocampus					0.03	0.01 to 0.05	3.37	<0.001	-0.02	-0.03 to 0	-1.78	0.08
L Hippocampus					0.05	0.03 to 0.07	4.75	<0.001	-0.01	-0.02 to 0.01	-0.84	0.4
R SFG					0.05	0.03 to 0.07	5.43	<0.001	-0.01	-0.03 to 0.01	1.05	0.29
L SFG					0.04	0.02 to 0.06	4.53	<0.001	-0.01	-0.02 to 0.01	-1.02	0.31
R DLPFC					0.06	0.04 to 0.07	6.57	<0.001	-0.01	-0.03 to 0	-1.42	0.16
L DLPFC					0.06	0.04 to 0.07	6.79	<0.001	-0.01	-0.02 to 0.01	-1.07	0.28
R DMPFC					0.05	0.03 to 0.06	5.52	<0.001	-0.01	-0.03 to 0	-1.6	0.11
L DMPFC					0.04	0.03 to 0.06	5.51	<0.001	-0.01	-0.03 to 0	1.59	0.11
**Model 2[b]**												
R Hippocampus	-0.03	-0.05 to -0.01	-2.71	0.01								
L Hippocampus	-0.02	-0.05 to -0.01	-2.23	0.03								
R SFG	-0.04	-0.06 to -0.02	-3.39	<0.001								
L SFG	-0.03	-0.05 to -0.01	-3.36	<0.001								
R DLPFC	-0.07	-0.09 to -0.05	-6.85	<0.001								
L DLPFC	-0.06	-0.08 to -0.04	-6.61	<0.001								
R DMPFC	-0.04	-0.06 to -0.02	-4.47	<0.001								
L DMPFC	-0.04	-0.05 to -0.02	-4.73	<0.001								
**Model 3[c]**												
R Hippocampus	<-0.01	-0.03 to 0.02	-0.17	0.87	0.03	0.01 to 0.05	2.54	0.01				
L Hippocampus	-0.01	-0.02 to 0.03	0.47	0.64	0.05	0.03 to 0.07	4.34	<0.001				
R SFG	-0.01	-0.03 to 0.02	-0.54	0.59	0.05	0.03 to 0.07	5.2	<0.001				
L SFG	-0.01	-0.03 to 0.02	-0.68	0.5	0.04	0.02 to 0.06	4.43	<0.001				
R DLPFC	-0.04	-0.06 to -0.02	-3.38	<0.001	0.05	0.03 to 0.07	5.26	<0.001				
L DLPFC	-0.03	-0.05 to -0.01	-2.95	<0.01	0.05	0.03 to 0.07	5.65	<0.001				
R DMPFC	-0.02	-0.04 to 0.01	-1.43	0.15	0.05	0.03 to 0.07	5.24	<0.001				
L DMPFC	-0.02	-0.04 to 0.01	-1.41	0.16	0.04	0.03 to 0.06	5.15	<0.001				

Abbreviations: *Std. b* = standardized coefficient estimates; PRFQ = Parent-Reported Financial Adversity Questionnaire; SFG = superior frontal gyrus; DLPFC = dorsal lateral prefrontal cortex; DMPFC = dorsal medial prefrontal cortex

[a] Model 1 included household income and PRFQ as predictors with age, sex, race/ethnicity, and intracranial volume included as covariates
[b] Model 2 included NP as a predictor, with age, sex, intracranial volume, and race/ethnicity included as covariates.
[c] Model 3 included household income, PRFQ, and NP as predictors with age, sex, intracranial volume, and race/ethnicity included as covariates.

© 2020 Taylor RL et al. *JAMA Network Open.*

eTable 3. Model Output for SEM Analyses Investigating Evidence for Future Mediation with Race/Ethnicity as a Covariate												
	**Path *a*** **NP→ Brain Factors**			**Path *b*** **Brain Factors →Cognitive Tasks**			***a x b*** **Indirect Effect**			***c'*** **NP →Cognitive Tasks with Brain Factors in Model**		
	**Estimate *(SE)***	***z***	***p***	**Estimate *(SE)***	***z***	***p***	**Estimate *(SE)***	***z***	***p***	**Estimate *(SE)***	***z***	***p***
**Picture Vocabulary**												
NP										-0.08 *(0.02)*	-4.97	<0.001
Prefrontal Factor	-0.23 *(0.01)*	-21.27	<0.001	0.1 *(0.02)*	5.17	<0.001	-0.02 *(0.01)*	-5.04	<0.001			
Hippocampal Factor	-0.19 *(0.01)*	-16.15	<0.001	0.06 *(0.02)*	3.18	<0.01	-0.01*(0.004)*	-3.12	<0.01			
**Oral Reading**												
NP										-0.03 *(0.02)*	-1.92	0.06
Prefrontal Factor	-0.23 *(0.01)*	-21.27	<0.001	0.06 *(0.02)*	2.96	<0.01	-0.01 *(0.01)*	-2.94	<0.01			
Hippocampal Factor	-0.19 *(0.01)*	-16.15	<0.001	0.06 *(0.02)*	3.26	<0.01	-0.01 *(0.004)*	-3.524	<0.01			
**Dimensional Card Sort**												
NP										-0.07 *(0.02)*	-4.58	<0.001
Prefrontal Factor	-0.23 *(0.01)*	-21.27	<0.001	0.05 *(0.02)*	2.37	0.02	-0.01 *(0.01)*	-2.35	0.02			
Hippocampal Factor	-0.19 *(0.01)*	-16.15	<0.001	0.03 *(0.02)*	1.52	0.13	-0.01*(0.004)*	-1.51	0.13			
**Flanker**												
NP										-0.07 *(0.02)*	-4.07	<0.001
Prefrontal Factor	-0.23 *(0.01)*	-21.27	<0.001	0.05 *(0.02)*	2.35	0.02	-0.01 *(0.01)*	-2.35	0.02			
Hippocampal Factor	-0.19 *(0.01)*	-16.15	<0.001	0.03 *(0.02)*	1.68	0.09	-0.01 *(0.004)*	-1.67	0.1			
**List Sorting**												
NP										-0.01 *(0.02)*	-4.93	<0.001
Prefrontal Factor	-0.23 *(0.01)*	-21.27	<0.001	0.09 *(0.02)*	4.42	<0.001	-0.02 *(0.01)*	-4.35	<0.001			
Hippocampal Factor	-0.19 *(0.01)*	-16.15	<0.001	0.01 *(0.02)*	0.37	0.71	-0.001 *(0.004)*	-0.37	0.71			
**Pattern Comparison**												
NP										-0.05 *(0.02)*	-3.08	<0.01
Prefrontal Factor	-0.23 *(0.01)*	-21.27	<0.001	0.02 *(0.02)*	1.12	0.26	-0.01 *(0.01)*	-1.12	0.23			
Hippocampal Factor	-0.19 *(0.01)*	-16.15	<0.001	0.02 *(0.02)*	0.178	0.44	-0.004 *(0.004)*	-0.78	0.44			
**Picture Sequence**												
NP										-0.02 *(0.02)*	-1.19	0.23
Prefrontal Factor	-0.23 *(0.01)*	-21.27	<0.001	0.03 *(0.02)*	1.39	0.17	-0.01 *(0.01)*	-1.39	0.17			
Hippocampal Factor	-0.19 *(0.01)*	-16.15	<0.001	0.05 *(0.02)*	2.71	<0.01	-0.01 *(0.004)*	-2.68	<0.01			

[a] Covariates included: age, sex, race/ethnicity, household income, and intracranial volume.

[b] Fit indices suggest that models were a good fit for the data: Prefrontal models (CFI = 0.936, RMSEA = 0.087 , SRMR = 0.071); Hippocampal models (CFI = 0.940, RMSEA = 0.084, SRMR = 0.053).

Note: NP was modeled as the predictor with prefrontal and hippocampal factors as mediators, with separate cognitive tasks as the outcome (7 models total).

© 2020 Taylor RL et al. *JAMA Network Open.*

eAppendix 2. *Sample Sizes Used for Each MLM Analysis*

eTable 4. Sample Sizes Used for Each MLM Analysis

NIH Toolbox-CB Measures	*n*	Brain ROIs	*n*
**Model 1[a]**			
**Picture Vocab.**	10618	**R Hippocampus**	10455
**Oral Reading**	10607	**L Hippocampus**	10455
**Dim. Card Sort**	10614	**R SFG**	10455
**Flanker**	10613	**L SFG**	10455
**List Sorting**	10579	**R DLPFC**	10455
**Pattern Comp.**	10598	**L DLPFC**	10455
**Picture Seq.**	10608	**R DMPFC**	10455
**Total Composite**	10390	**L DMPFC**	10455
**Model 2[b]**			
**Picture Vocab.**	11031	**R Hippocampus**	10844
**Oral Reading**	11019	**L Hippocampus**	10844
**Dim. Card Sort**	11027	**R SFG**	10844
**Flanker**	11026	**L SFG**	10844
**List Sorting**	10991	**R DLPFC**	10844
**Pattern Comp.**	11010	**L DLPFC**	10844
**Picture Seq.**	11022	**R DMPFC**	10844
**Total Composite**	10789	**L DMPFC**	10844
**Model 3[c]**			
**Picture Vocab.**	10030	**R Hippocampus**	9965
**Oral Reading**	10020	**L Hippocampus**	9965
**Dim. Card Sort**	10027	**R SFG**	9965
**Flanker**	10026	**L SFG**	9965
**List Sorting**	9999	**R DLPFC**	9965
**Pattern Comp.**	10013	**L DLPFC**	9965
**Picture Seq.**	10022	**R DMPFC**	9965
**Total Composite**	9812	**L DMPFC**	9965

© 2020 Taylor RL et al. *JAMA Network Open.*

eAppendix 3. ***Missingness Within the Study Sample and Comparison of Missing Values Among Demographic and Outcome Variables***

eTable 5. Missingness of Data in Key Model Variables

Variable	*Missing Cases*
**Age**	12
**Sex**	13
**Household Income**	1020
**Neighborhood Poverty**	695
**Brain ROIs (Regions of Interest)**	339
**Picture Vocab.**	155
**Oral Reading**	169
**Dim. Card Sort**	160
**Flanker**	161
**List Sorting**	204
**Pattern Comp.**	179
**Picture Seq.**	167
**Total Composite**	410

© 2020 Taylor RL et al. *JAMA Network Open.*

eFigure 3. Comparison of Missing Values Among Demographic Variables

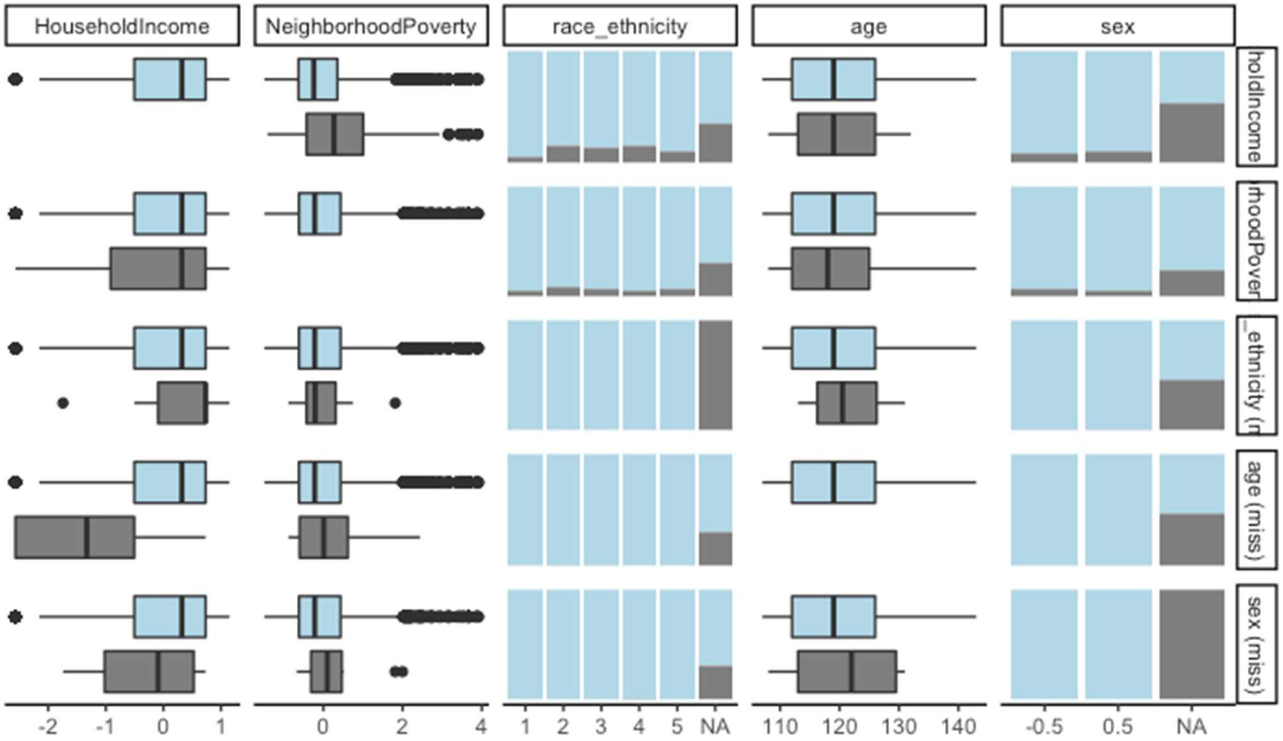

The GGally() and finalfit() packages were used to determine whether missingness of key predictor variables (NP and household income) was significantly related to missingness of demographic variables and whether non-completion of demographic and outcome variables was related to certain levels of NP and household income. Missingness of key predictor variables (household income and neighborhood poverty) did suggest some association with missingness of other demographic variables (race/ethnicity/sex).

Note: Boxplots are comparing the distributions of missing data (gray) to non-missing data (blue) across variables

© 2020 Taylor RL et al. *JAMA Network Open.*

eFigure 4. Comparison of Missing Values Among Brain ROIs

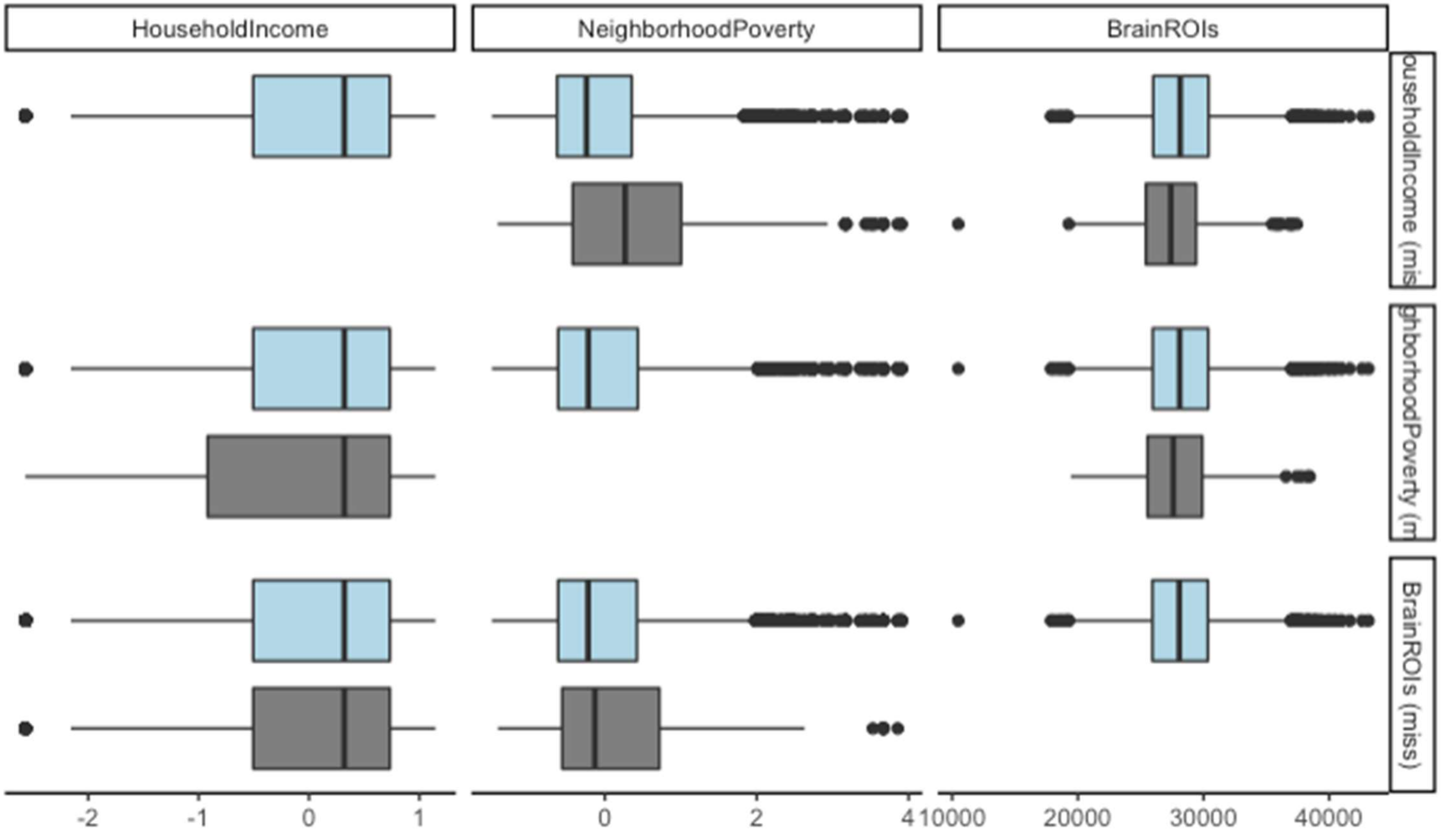

The GGally and finalfit() packages in R were used to visualize and determine whether missingness of key brain regions of interest (dlPFC, dmPFC, SFG) was related to certain levels of NP and household income. The visualization does not indicate that missingness of brain variables is based on certain levels of NP and household income**.**

Note: Boxplots are comparing the distributions of missing data (gray) to non-missing data (blue) across variables

© 2020 Taylor RL et al. *JAMA Network Open.*

eFigure 5. Comparison of Missing Values Among Cognitive Measures

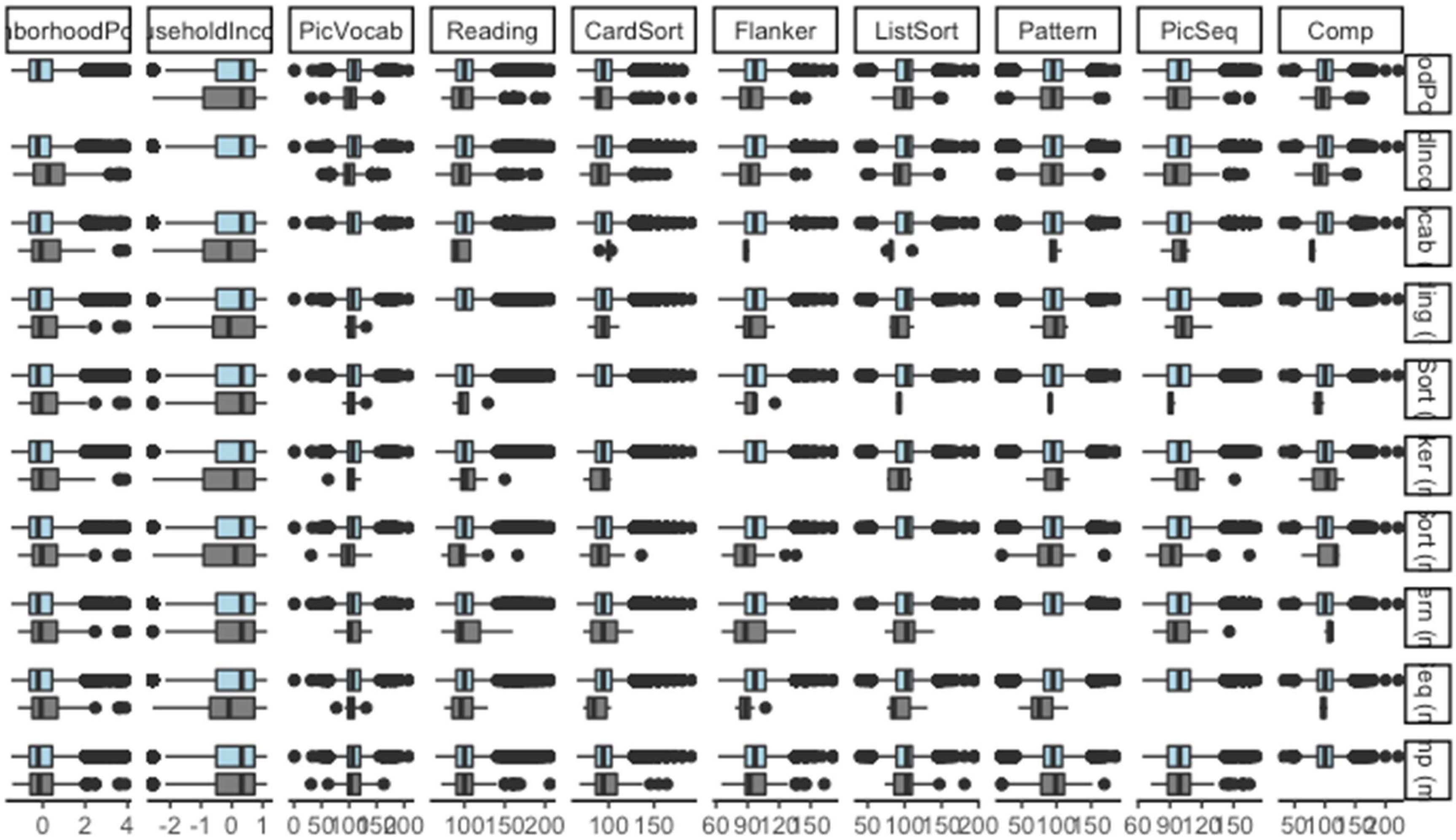

The GGally() and finalfit() packages in R were used to visualize and determine whether missingness of cognitive measures (Picture Vocabulary, Oral Reading, Dimensional Card Sort, Flanker Inhibitory Control, List Sorting Working Memory, Pattern Comparison, Picture Sequence, and Total Composite). was related to certain levels of NP and household income. The visualization does not indicate that missingness of cognitive variables is based on certain levels of NP and household income

Note: Boxplots are comparing the distributions of missing data (gray) to non-missing data (blue) across variables

eAppendix 4. Distribution of NP and Household Income in Overall Sample and Additional Information on Measure of NP, PRFQ, and Household Income

Residential History Questionnaire and Area Deprivation Index*:* The ABCD Study uses the primary current home address provided by the parent via the Residential History Questionnaire. This address is used to generate the Area Deprivation Index (ADI) for the census tract that contains this address. The census data that is used here is from the 2010 Census and some supplementary American Community Survey information. The ADI consists of 17 census variables that use census tracts to reference different aspects of SES. The original factor analysis on the ADI yielded a single factor with the lowest loading of 0.45 (percent of occupied houses with complete plumbing)[34]. A factor analysis of these same variables in the current data set also supported a single factor, with the lowest factor loading of 0.21 for the same variable (percent of occupied houses with complete plumbing). However, the factor loadings of some of the variables were low and seemed to reflect geographical differences in cost-of-living across sites that were less indicative of objective disadvantage (e.g., median mortgage or rent costs) or less modern disadvantage indices (e.g. percentage of homes without a telephone). Thus, to create a homogenous measure of neighborhood poverty, a cutoff of 0.6 loading was used for inclusion of variables in the final measure. Nine of the 17 census variables survived this cutoff (see eTable 6 for list of all 17 variables). Each of the nine retained variables were z-scored and averaged, with items reverse-scored where appropriate (% of individuals ≥ 25 with a high school diploma, percentage of homeowners, & median family income) to ensure the same directionality for all variables. A higher score on this variable indicated greater neighborhood poverty.

PRFQ: The items included on the Parent-Reported Financial Adversity Questionnaire (PRFQ) questionnaire are shown in eTable 7. PRFQ indexes self-report of finances that may better account for the association of income level to area cost-of-living.

© 2020 Taylor RL et al. *JAMA Network Open.*

Household income: Self -report of combined income of all members of household over the past 12 months, ranging from 1 (<$5,000) to 10 ($200,000 or greater). Median in the current sample is 8 ($75,000 - $99,000). The measure of household income came from the General Social Survey (Smith et al. 2015) and is the measure recommended by the National Institute of Mental Health (Barch et al. 2016).

© 2020 Taylor RL et al. *JAMA Network Open.*

eTable 6. Factor Loadings of ADI Variables in ABCD Sample

Census Variable	Factor Loading
**Percentage of labor force aged > 16 unemployed[a]**	**0.76**
Percentage of persons aged >= 16 employed in white collar positions	-0.46
**Percentage of families below poverty level**	**0.95**
**Percentage of population below 138% of the poverty threshold**	**0.98**
Percentage of crowding	0.27
**Percentage of single parent households**	**0.84**
**Percentage of occupied housing units without a motor vehicle**	**0.73**
**Percentage of population aged >/= 25 with at least a high school diploma[b]**	**-0.74**
Percentage of population aged >/= 25 with < 9 years of education	0.59
**Percentage of home owners**	**-0.77**
**Median family income**	**-0.77**
**Income disparity**	**0.87**
Median home value	-0.54
Median monthly mortgage	0.58
Percentage of occupied units without complete plumbing	0.21
Percentage of occupied units without a telephone	0.35
Median gross rent	0.59

[a] Variables in bold were included in the Neighborhood Poverty measure

[b] Percentage of pop. with high school diploma, percentage of homeowners, and median family income were reverse scored.

Abbreviations: ADI, Area Deprivation Index.

© 2020 Taylor RL et al. *JAMA Network Open.*

eTable 7. Parent-Reported Financial Adversity Questionnaire (PRFQ) Items
Needed food but couldn't afford to buy it or couldn't afford to go out to get it?
Didn't pay the full amount of the rent or mortgage because you could not afford it?
Were evicted from your home for not paying the rent or mortgage?
Were without telephone service because you could not afford it?
Had services turned off by the gas or electric company, or the oil company wouldn't deliver oil because payments were not made?
Had someone who needed to see a doctor or go to the hospital but didn't go because you could not afford it?
Had someone who needed a dentist but couldn't go because you could not afford it?

© 2020 Taylor RL et al. *JAMA Network Open.*

eFigure 6. Distribution of Neighborhood Poverty and Household Income in the Current Sample

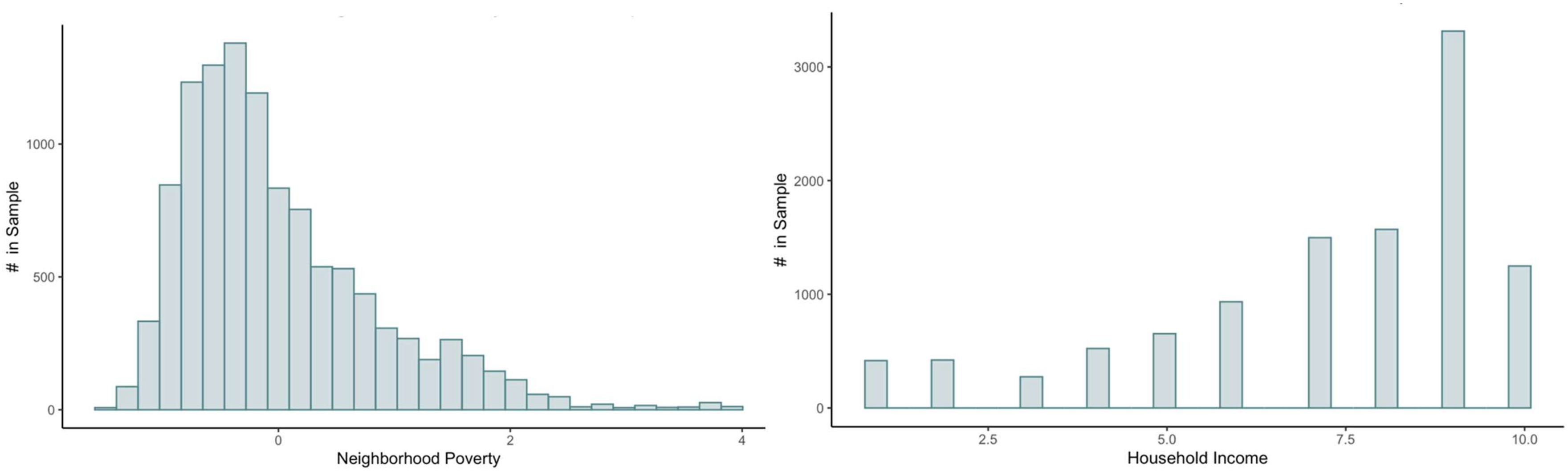

Mdn = -0.22
Range = 5.4 (min = -1.49, max = 3.91)

Mdn = 8
Range = 9 (min = 1, max = 10)

© 2020 Taylor RL et al. *JAMA Network Open.*

eAppendix 5. MLM Results Tables With ADI Weighted Sum as Independent Variable

eTable 8. Model Output for NIH Toolbox-CB Measures with ADI weighted sum

	**ADI Sum**				**Household Income**				**PRFQ**			
**NIH Toolbox-CB Measures**	**Std. b**	**CIs**	***t***	***p***	**Std. b**	**CIs**	***t***	***p***	**Std. b**	**CIs**	***t***	***p***
**Model 1[a]**												
Picture Vocab.					0.36	0.34 to 0.38	34.82	<0.001	-0.04	-0.06 to -0.02	-2.63	<0.01
Oral Reading					0.28	0.26 to 0.3	25.89	<0.001	-0.04	-0.06 to -0.02	-3.52	<0.001
Dim. Card Sort					0.18	0.16 to 0.2	16.7	<0.001	-0.03	-0.06 to -0.01	-3.08	<0.01
Flanker					0.16	0.14 to 0.19	15.18	<0.001	0.00	-0.02 to 0.02	-0.2	0.84
List Sorting					0.28	0.26 to 0.31	26.73	<0.001	-0.02	-0.04 to 0	-1.62	0.11
Pattern Comp.					0.11	0.09 to 0.13	9.93	<0.001	-0.02	-0.04 to 0	-1.95	0.05
Picture Seq.					0.18	0.16 to 0.2	16.75	<0.001	-0.03	-0.05 to -0.01	-2.76	0.01
Total Composite					0.38	0.36 to 0.4	36.91	<0.001	-0.05	-0.07 to -0.03	-4.35	<0.001
**Model 2[b]**												
Picture Vocab.	-0.2	-0.22 to -0.19	-20.98	<0.001								
Oral Reading	-0.18	-0.2 to -0.16	-18.64	<0.001								
Dim. Card Sort	-0.12	-0.13 to -0.1	-12.34	<0.001								
Flanker	-0.11	-0.13 to -0.09	-11.5	<0.001								
List Sorting	-0.17	-0.19 to -0.15	-17.29	<0.001								
Pattern Comp.	-0.09	-0.11 to -0.07	-9.58	<0.001								
Picture Seq.	-0.09	-0.11 to -0.08	-9.74	<0.001								
Total Composite	-0.24	-0.26 to -0.22	-24.56	<0.001								
**Model 3[c]**												
Picture Vocab.	-0.07	-0.09 to -0.05	-7.14	<0.001	0.34	0.31 to 0.36	29.92	<0.001	-0.04	-0.06 to -0.02	-3.81	<0.01
Oral Reading	-0.09	-0.11 to -0.06	-7.89	<0.001	0.25	0.23 to 0.27	21.48	<0.001	-0.04	-0.06 to -0.01	-3.23	<0.01
Dim. Card Sort	-0.05	-0.07 to -0.03	-4.55	<0.001	0.17	0.14 to 0.19	14.11	<0.001	-0.03	-0.05 to -0.01	-2.764	0.01
Flanker	-0.06	-0.08 to -0.04	-5.5	<0.001	0.15	0.12 to 0.17	12.5	<0.001				
List Sorting	-0.07	-0.09 to -0.05	-6.76	<0.001	0.26	0.24 to 0.28	22.71	<0.001				
Pattern Comp.	-0.06	-0.08 to -0.03	-5.43	<0.001	0.09	0.07 to 0.11	7.71	<0.001				
Picture Seq.	-0.03	-0.05 to -0.01	-2.31	0.02	0.17	0.15 to 0.19	14.4	<0.001	-0.03	-0.05 to -0.01	-2.47	0.01
Total Composite	-0.11	-0.13 to -0.08	-10.2	<0.001	0.35	0.33 to 0.37	31.18	<0.001	-0.04	-0.06 to -0.02	-3.74	<0.01

[a] Model 1 included household income and PRFQ as predictors with age and sex included as covariates.
[b] Model 2 included ADI weighted sum as a predictor with age and sex included as covariates. 0.33 to 0.37
[c] Model 3 included household income, PRFQ, and ADI weighted sum as predictors with age and sex included as covariates.

© 2020 Taylor RL et al. *JAMA Network Open.*

**eTable 9. Model Output for Brain Regions of Interest (ROIs) with ADI weighted sum**												
	**ADI Sum**				**Household Income**				**PRFQ**			
**Brain ROIs**	**Std. b**	**CIs**	***t***	***p***	**Std. b**	**CIs**	***t***	***p***	**Std. b**	**CIs**	***t***	***p***
**Model 1[a]**												
R Hippocampus					0.06	0.05 to 0.08	7.16	<0.001	-0.02	-0.04 to -0.01	-2.63	0.01
L Hippocampus					0.07	0.05 to 0.09	7.73	<0.001	-0.01	-0.03 to 0.01	-1.32	0.19
R SFG					0.1	0.08 to 0.12	11.35	<0.001	-0.02	-0.03 to 0	-1.78	0.09
L SFG					0.09	0.07 to 0.1	10.22	<0.001	-0.01	-0.03 to 0	-1.73	0.09
R DLPFC					0.12	0.1 to 0.13	14.58	<0.001	-0.02	-0.03 to 0	-2.35	0.03
L DLPFC					0.11	0.1 to 0.13	14.39	<0.001	-0.01	-0.03 to 0	-1.93	0.07
R DMPFC					0.1	0.08 to 0.12	12.56	<0.001	-0.02	-0.04 to -0.01	-2.68	0.01
L DMPFC					0.1	0.08 to 0.11	12.25	<0.001	-0.02	-0.03 to 0	-2.5	0.01
**Model 2[b]**												
R Hippocampus	-0.04	-0.05 to -0.02	-4.94	<0.001								
L Hippocampus	-0.03	-0.05 to -0.02	-3.9	<0.001								
R SFG	-0.06	-0.07 to -0.04	-7.21	<0.001								
L SFG	-0.05	-0.07 to -0.04	-6.86	<0.001								
R DLPFC	-0.07	-0.09 to -0.06	-10.22	<0.001								
L DLPFC	-0.06	-0.07 to -0.05	-8.89	<0.001								
R DMPFC	-0.07	-0.08 to -0.05	-9.55	<0.001								
L DMPFC	-0.05	-0.07 to -0.04	-8.28	<0.001								
**Model 3[c]**												
R Hippocampus	-0.02	-0.03 to 0	-1.5	0.13	0.06	0.04 to 0.08	5.83	<0.001	-0.02	-0.04 to -0.01	-2.63	0.01
L Hippocampus	-0.01	-0.02 to 0.01	-0.77	0.44	0.07	0.05 to 0.09	7.86	<0.001				
R SFG	-0.02	-0.04 to -0.01	-2.21	0.03	0.09	0.08 to 0.11	11.13	<0.001				
L SFG	-0.02	-0.04 to -0.01	-2.28	0.02	0.09	0.07 to 0.1	10.13	<0.001				
R DLPFC	-0.03	-0.05 to -0.02	-4	<0.001	0.11	0.09 to 0.12	12.37	<0.001	-0.01	-0.03 to 0	-1.8	0.07
L DLPFC	-0.02	-0.03 to -0.01	-2.64	<0.01	0.1	0.09 to 0.12	12.64	<0.001				
R DMPFC	-0.03	-0.05 to -0.02	-3.95	<0.001	0.09	0.07 to 0.11	10.72	<0.001	-0.01	-0.03 to 0	-1.75	0.08
L DMPFC	-0.02	-0.03 to -0.01	-2.61	0.01	0.09	0.08 to 0.11	7.25	<0.001	-0.01	-0.03 to 0	-1.69	0.07

[a] Model 1 included household income and PRFQ as predictors with age, sex, and intracranial volume included as covariates
[b] Model 2 included ADI weighted sum as a predictor, with age, sex, and intracranial volume included as covariates.
[c] Model 3 included household income, PRFQ, and ADI weighted sum as predictors with age, sex, and intracranial volume included as covariates.

© 2020 Taylor RL et al. *JAMA Network Open.*

eAppendix 6. FreeSurfer QC Methods

Quality control of the data was performed using both manual and automated processes. Trained technicians reviewed all images to check for artifacts and irregularities. The cortical reconstruction of post-processed images was double-rated on a scale from 0-3, with 3 indicating the greatest amount of artifact. Technicians gave recommendations for usability based on scores. Automated processes were used to calculate signal-to-noise-ratio (SNR) and head motion values[36]. See Hagler et al. for more information regarding preprocessing and specific QC procedures.

© 2020 Taylor RL et al. *JAMA Network Open.*

eAppendix 7. ML Model 3 Results Using Only Those Participants With a 0 on the Artifact Score

eTable 10. Model 3 Output for Brain Regions of Interest (ROIs) Using Participants with Zero Artifact Score

	**Neighborhood Poverty**				**Household Income**				**PRFQ**			
**Brain ROIs**	**Std. b**	**CIs**	***t***	***p***	**Std. b**	**CIs**	***t***	***p***	**Std. b**	**CIs**	***t***	***p***
**Model 3[c]**												
R Hippocampus	-0.04	-0.08 to -0.01	-2.27	0.04	0.05	0.02 to 0.08	3.13	<0.010	-0.02	-0.05 to 0.01	-1.27	0.246
L Hippocampus	-0.01	-0.05 to 0.03	-0.47	0.67	0.08	0.05 to 0.12	4.96	<0.001	0	-0.03 to 0.03	-0.08	0.939
R SFG	-0.06	-0.07 to -0.01	-2.06	0.07	0.07	0.04 to 0.1	4.62	<0.001	-0.01	-0.04 to 0.01	-0.89	0.406
L SFG	-0.04	-0.07 to -0.01	-1.99	0.05	0.07	0.03 to 0.1	4.18	<0.001	-0.02	-0.05 to 0.01	-1.57	0.156
R DLPFC	-0.09	-0.11 to -0.06	-5.54	<0.001	0.07	0.05 to 0.1	5.33	<0.001	-0.01	-0.03 to 0.01	-0.96	0.389
L DLPFC	-0.07	-0.1 to -0.05	-5.07	<0.001	0.07	0.04 to 0.09	5.12	<0.001	-0.01	-0.04 to 0.01	-1.36	0.215
R DMPFC	-0.06	-0.09 to -0.03	-3.64	<0.01	0.06	0.03 to 0.09	4.36	<0.001	-0.02	-0.05 to 0	-2.00	0.065
L DMPFC	-0.04	-0.07 to -0.01	-2.93	0.01	0.06	0.03 to 0.08	4.25	<0.001	-0.02	-0.04 to 0	-2.02	0.065

[a] Model 3 included household income, PRFQ, and NP as predictors with age, sex, and intracranial volume included as covariates.

© 2020 Taylor RL et al. *JAMA Network Open.*

eAppendix 8. NIH Toolbox-CB and Brain ROI EFA

eTable 11. Results of EFA with NIH Toolbox-CB				
**Brain ROI**	**Factor 1**	**Factor 2**	**Factor 3**	**Factor 4**
Card Sort	0.71		0.11	0.11
Flanker	0.66	0.10		
Pattern Comp.	0.77			
Oral Reading	0.13	0.95	0.24	
Picture Vocab	0.13	0.25	0.95	
Picture Seq.	0.15		0.10	0.98
List Sorting	0.46	0.24	0.26	0.22
**SS Loadings**	2.16	1.35	1.32	1.13
**Proportion Var**	0.27	0.17	0.17	0.14
**Cumulative Var**	0.27	0.44	0.60	0.75

© 2020 Taylor RL et al. *JAMA Network Open.*

eTable 12. Results of EFA with Brain ROIs				
**Brain ROI**	**Factor 1**	**Factor 2**	**Factor 3**	**Factor 4**
R Hippocampus	0.28	0.87	0.15	
L Hippocampus	0.28	0.81	0.14	
R SFG	0.82	0.23	0.23	-0.16
L SFG	0.75	0.21	0.61	
R DLPFC	0.88	0.28		
L DLPFC	0.91	0.31		0.27
R DMPFC	0.92	0.29	0.16	-0.19
L DMPFC	0.87	0.31	0.29	
**SS Loadings**	4.62	1.86	0.59	0.15
**Proportion Var**	0.51	0.21	0.07	0.02
**Cumulative Var**	0.51	0.72	0.79	0.8

© 2020 Taylor RL et al. *JAMA Network Open.*

eAppendix 9. MLM Unstandardized Results Tables

eTable 13. Unstandardized Model Output for NIH Toolbox-CB Measures

	Neighborhood Poverty				Household Income				PRFQ			
**NIH Toolbox-CB Measures**	***b***	**CIs**	***t***	***p***	***b***	**CIs**	***t***	***p***	***b***	**CIs**	***t***	***p***
**Model 1[a]**												
Picture Vocab.					2.54	2.39 to 2.68	34.82	<0.001	-0.68	-1 to -0.37	-2.63	0.001
Oral Reading					2.23	2.05 to 2.38	25.89	<0.001	-0.66	-1.03 to -0.29	-3.52	<0.001
Dim. Card Sort					1.13	1 to 1.27	16.7	<0.001	-0.46	-0.76 to -0.17	-3.08	<0.01
Flanker					9.28	0.81 to 1.05	15.18	<0.001	-0.03	-0.04 to 0.02	-0.2	0.84
List Sorting					1.74	1.61 to 1.86	26.73	<0.001	-0.23	-0.51 to 0.05	-1.62	0.11
Pattern Comp.					0.99	0.79 to 1.18	9.93	<0.001	-0.43	-0.88 to 0	-1.95	0.05
Picture Seq.					1.21	1.06 to 1.35	16.75	<0.001	-0.44	-0.75 to -0.13	-2.76	0.01
Total Composite					2.84	2.69 to 2.99	36.91	<0.001	-0.74	-1.07 to -0.41	-4.35	<0.001
**Model 2[b]**												
Picture Vocab.	-6.77	-7.14 to -6.4	-35.85	<0.001								
Oral Reading	-5.11	-5.54 to -4.68	-23.3	<0.001								
Dim. Card Sort	-3.39	-3.72 to -3.04	-19.65	<0.001								
Flanker	-2.54	-2.84 to -2.23	-16.26	<0.001								
List Sorting	-4.36	-4.68 to -4.03	-26.11	<0.001								
Pattern Comp.	-3.19	-3.68 to -2.69	-12.71	<0.001								
Picture Seq.	-3.07	-3.43 to -2.71	-16.66	<0.001								
Total Composite	-7.41	-7.81 to -7.02	-37.05	<0.001								
**Model 3[c]**												
Picture Vocab.	-3.07	-3.55 to -2.58	-12.43	<0.001	1.94	1.77 to 2.12	21.98	<0.001	-0.67	-0.92 to -0.28	-3.63	0.11
Oral Reading	-1.33	-1.9 to -0.76	-4.58	<0.001	1.95	1.74 to 2.15	18.72	<0.001	-0.67	-1.05 to -0.29	-3.44	0.11
Dim. Card Sort	-1.79	-2.24 to -1.33	-7.72	<0.001	0.8	0.64 to 0.96	9.63	<0.001	-0.43	-0.73 to -0.12	-2.76	0.21
Flanker	-1.32	-1.73 to -0.91	-6.32	<0.001	0.69	0.54 to 0.83	9.19	<0.001				
List Sorting	-1.9	-2.33 to -1.47	-8.62	<0.001	1.38	1.22 to 1.53	17.4	<0.001				
Pattern Comp.	-2.08	-2.74 to -1.42	-6.16	<0.001	0.61	0.37 to 0.85	5.03	<0.001				
Picture Seq.	-1.19	-1.67 to -0.71	-4.84	<0.001	0.95	0.78 to 1.13	10.8	<0.001	-0.65	-0.71 to -0.44	-2.36	0.12
Total Composite	-3.22	-3.73 to -2.71	-12.44	<0.001	2.22	2.04 to 2.41	23.92	<0.001	-0.66	-1 to -0.31	-3.71	0.12

Abbreviation: *b* = unstandardized coefficient estimates; PRFQ = Parent-Reported Financial Adversity Questionnaire

[a] Model 1 included household income and PRFQ as predictors with age, sex, and race/ethnicity included as covariates.
[b] Model 2 included NP as a predictor with age, sex, and race/ethnicity included as covariates.
[c] Model 3 included household income, PRFQ, and NP as predictors with age, sex, and race/ethnicity included as covariates.

© 2020 Taylor RL et al. *JAMA Network Open.*

eTable 14. Unstandardized Model Output for Brain Regions

	Neighborhood Poverty				Household Income				PRFQ			
**Brain ROIs**	***b***	**CIs**	***t***	***p***	***b***	**CIs**	***t***	***p***	***b***	**CIs**	***t***	***p***
**Model 1[a]**												
R Hippocampus					11.28	8.19 to 14.37	7.16	<0.001	-8.95	-15.62 to -2.28	-2.63	0.01
L Hippocampus					12.71	9.49 to 15.93	7.73	<0.001	-4.71	-11.67 to 2.26	-1.32	0.19
R SFG					134.1	110.9 to 157.3	11.35	<0.001	-45.4	-95.5 to 4.66	-1.78	0.09
L SFG					117.7	95.15 to 140.3	10.22	<0.001	-43.1	-91.9 to 5.67	-1.73	0.09
R DLPFC					0.011	0.001 to 0.013	14.58	<0.001	-0.003	-0.007 to 0	-2.35	0.03
L DLPFC					0.01	0.001 to 0.011	14.39	<0.001	-0.002	-0.005 to 0	-1.93	0.07
R DMPFC					0.007	0.006 to 0.008	12.56	<0.001	-0.003	-0.005 to 0	-2.68	0.01
L DMPFC					0.006	0.005 to 0.007	12.25	<0.001	-0.003	-0.005 to -0.007	-2.5	0.01
**Model 2[b]**												
R Hippocampus	-33.42	-41.26 to -25.57	-8.35	<0.001								
L Hippocampus	-28.51	-36.77 to -20.26	-6.77	0.04								
R SFG	-345.8	-404.8 to -286.9	-11.5	<0.001								
L SFG	-320.8	-378.1 to -263.5	-10.97	<0.001								
R DLPFC	-0.032	-0.04 to -0.028	-17.5	<0.001								
L DLPFC	-0.03	-0.033 to -0.027	-16.75	<0.001								
R DMPFC	-0.02	-0.022 to -0.017	-14.21	<0.001								
L DMPFC	-0.019	-0.021 to -0.016	-13.98	<0.001								
**Model 3[c]**												
R Hippocampus	-15.36	-25.78 to -4.94	-2.89	0.01	7.89	4.16 to 11.63	2.54	0.01	-5.46	-16.11 to -2.26	-2.6	0.05
L Hippocampus	-7.19	-18.08 to 3.7	-1.29	0.21	11.07	7.16 to 14.97	4.34	<0.001				
R SFG	-169.4	-246.9 to -91.9	-4.24	<0.001	108.1	81.8 to 134.3	5.2	<0.001				
L SFG	-166.7	-242.1 to 91.2	-4.29	<0.001	93.8	68.2 to 119.4	4.43	<0.001				
R DLPFC	-0.021	-0.025 to -0.016	-8.43	<0.001	0.007	0.005 to 0.009	5.26	<0.001	-0.003	-0.006 to -0.001	-1.68	0.09
L DLPFC	-0.018	-0.023 to -0.014	-7.7	<0.001	0.007	0.005 to 0.009	5.65	<0.001				
R DMPFC	-0.011	-0.015 to -0.008	-6.08	<0.001	0.005	0.004 to 0.006	5.24	<0.001	-0.002	-0.004 to 0	-1.72	0.12
L DMPFC	-0.01	-0.014 to -0.006	-5.88	<0.001	0.005	0.003 to 0.006	5.15	<0.001	-0.002	-0.004 to 0	-1.69	0.12

Abbreviations: *b* = unstandardized coefficient estimates; PRFQ = Parent-Reported Financial Adversity Questionnaire; SFG = superior frontal gyrus; DLPFC = dorsal lateral prefrontal cortex; DMPFC = dorsal medial prefrontal cortex

[a] Model 1 included household income and PRFQ as predictors with age, sex, race/ethnicity, and intracranial volume included as covariates
[b] Model 2 included NP as a predictor, with age, sex, intracranial volume, and race/ethnicity included as covariates.
[c] Model 3 included household income, PRFQ, and NP as predictors with age, sex, intracranial volume, and race/ethnicity included as covariates.

© 2020 Taylor RL et al. *JAMA Network Open.*

eAppendix 10. MLM Results Looking at Orbital Frontal Regions as the Outcome Variable

eTable 15. Model Output for Orbital Frontal Cortex

	Neighborhood Poverty				Household Income				PRFQ			
**Brain ROIs**	**Std. b**	**CIs**	***t***	***p***	**Std. b**	**CIs**	***t***	***p***	**Std. b**	**CIs**	***t***	***p***
**Model 1[a]**												
R OFC					0.07	0.03 to 0.1	3.87	<0.001	-0.03	-0.06 to 0.01	-1.46	0.15
L OFC					0.07	0.04 to 0.1	6.72	<0.001	-0.03	-0.06 to 0	-1.92	0.06
**Model 2[b]**												
R OFC	-0.08	-0.12 to -0.05	-4.3	<0.001								
L OFC	-0.09	-0.12 to -0.05	-4.99	<0.001								
**Model 3[c]**												
R OFC	-0.05	-0.1 to 0	-1.92	0.06	0.06	0.02 to 0.1	3.21	<0.01				
L OFC	-0.04	-0.08 to 0	-1.89	0.06	0.06	0.03 to 0.1	3.66	<0.01				

[a] Model 1 included household income and PRFQ as predictors
[b] Model 2 included NP as a predictor, with age, sex, and intracranial volume included as covariates.
[c] Model 3 included household income, PRFQ, and NP as predictors with age, sex, and intracranial volume included as covariates.

© 2020 Taylor RL et al. *JAMA Network Open.*
